# Expression Profile of Ectopic Olfactory Receptors Determined by Deep Sequencing

**DOI:** 10.1371/journal.pone.0055368

**Published:** 2013-02-06

**Authors:** Caroline Flegel, Stavros Manteniotis, Sandra Osthold, Hanns Hatt, Günter Gisselmann

**Affiliations:** Department of Cell Physiology, Ruhr-University Bochum, Bochum, Germany; Monell Chemical Senses Center, United States of America

## Abstract

Olfactory receptors (ORs) provide the molecular basis for the detection of volatile odorant molecules by olfactory sensory neurons. The OR supergene family encodes G-protein coupled proteins that belong to the seven-transmembrane-domain receptor family. It was initially postulated that ORs are exclusively expressed in the olfactory epithelium. However, recent studies have demonstrated ectopic expression of some ORs in a variety of other tissues. In the present study, we conducted a comprehensive expression analysis of ORs using an extended panel of human tissues. This analysis made use of recent dramatic technical developments of the so-called Next Generation Sequencing (NGS) technique, which encouraged us to use open access data for the first comprehensive RNA-Seq expression analysis of ectopically expressed ORs in multiple human tissues. We analyzed mRNA-Seq data obtained by Illumina sequencing of 16 human tissues available from Illumina Body Map project 2.0 and from an additional study of OR expression in testis. At least some ORs were expressed in all the tissues analyzed. In several tissues, we could detect broadly expressed ORs such as OR2W3 and OR51E1. We also identified ORs that showed exclusive expression in one investigated tissue, such as OR4N4 in testis. For some ORs, the coding exon was found to be part of a transcript of upstream genes. In total, 111 of 400 OR genes were expressed with an FPKM (fragments per kilobase of exon per million fragments mapped) higher than 0.1 in at least one tissue. For several ORs, mRNA expression was verified by RT-PCR. Our results support the idea that ORs are broadly expressed in a variety of tissues and provide the basis for further functional studies.

## Introduction

Olfactory receptors (ORs) detect volatile odorant molecules in the environment. In 1991, Linda Buck and Richard Axel identified a supergene family that encodes G-protein coupled receptor proteins (GPCRs) in olfactory epithelium of the rat [1]. These authors postulated that ORs are exclusively expressed in the olfactory epithelium, where they are located in the cilia of olfactory sensory neurons. In 1998, Zhao et al. showed that ORs serve as neuronal odorant sensors [2]. The superfamily of ORs in humans represents the largest gene family known; about it contains approximately 400 functional OR genes and approximately 600 non-functional OR pseudogenes [3], [4]. OR genes are found throughout the human genome except on chromosome 20 and the Y chromosome [4], [5]. They are usually organized in clusters that are found mostly in telomeric regions [6]. Most OR genes have an intron-free reading frame of approximately 1000 nucleotides that encodes ∼330 amino acids [1], [5], [7]. Some ORs, including MHC-linked ORs, have splice variants in the 5′ untranslated gene regions (5′UTRs), suggesting that the transcription of these genes involves an unusual and complex regulatory mechanism [8], [9].

Recent studies have shown that expression of receptors encoded by the OR supergene family is not restricted to the olfactory epithelium. In 1992, only one year after the discovery of ORs, Parmentier and colleagues reported that mammalian ORs are also expressed in a non-olfactory tissue (testis) [10]. Later, Spehr and colleagues demonstrated the functional expression of an OR in human spermatozoa [11]. Activation of OR1D2 in spermatozoa by the odorant bourgeonal influences the swimming direction and swimming speed of spermatozoa. The expression of more than 50 ORs has been detected in the testes of several species including human, dog, mouse and rat [10]–[14]. The well characterized OR51E2, also known as PSGR, is highly expressed in prostate [15], [16]. Activation of this receptor by its specific ligand inhibits the proliferation of prostate cancer cells [17]. Moreover, odorants reaching the luminal environment of the gut may stimulate serotonin release via ORs present in enterochromaffin cells [18]. These examples illustrate the importance and some of the possible function of ORs outside the olfactory epithelium. Although many past studies have focused on the expression of ORs, most have only studied expression in a single mammalian tissue. Expression of individual OR transcripts in various tissues, including the autonomic nervous system [19], brain [20]–[22], tongue [23], [24], erythroid cells [25], prostate [15], placenta [26], gut [18] and kidney [27], has been described. So far, only a few systematic studies have analyzed the entire olfactory subtranscriptome in a variety of different human tissues. These studies were performed using EST data and microarray analysis [28], [29]. The results of these studies do not correlate well with each other, and only a few human ORs were detected using either approach [29].

The new high-throughput mRNA sequencing (RNA-Seq) technique, which is based on Next Generation Sequencing (NGS), has emerged as one of the most promising new developments in quantitative expression profiling [30], [31]. RNA-Seq provides high-resolution measurement of expression and is a suitable method for comprehensive expression analysis. One advantage of NGS is that it makes it possible to reanalyze existing sequencing data for a variety of purposes, such as the detection of novel transcribed gene loci and splice variants. In addition, NGS techniques allow comparison of data from different studies [32]. In recent years, the transcriptomes of a variety of tissues have been sequenced, and transcriptome data have accumulated in public data bases. Recently, RNA-Seq data sets of 16 different human tissues were generated as part of the Illumina Body Map project 2.0 (http://www.illumina.com). We reanalyzed these large data sets to establish a comprehensive overview of ectopic OR gene expression in multiple human tissues. In a subsequent experiment, expression of the most interesting ORs was verified by RT-PCR.

## Results

Due to the recent dynamic development of the NGS technique, a large number of transcriptome data sets obtained from multiple projects have accumulated. Several groups have already reanalyzed these data for a variety of purposes [33]–[35]. In our study, we reanalyzed the available RNA-Seq data from human tissues to assess the expression of OR genes in a broad range of tissues. The Illumina Body Map project 2.0 is a comprehensive human transcriptome project. We investigated sequence data for 16 different human tissues generated within this project and analyzed the 75-bp single-read data sets, which typically consist of ∼80 million reads. The large number of sequences produced by the HiSeq2000 Genome Analyzer provide for very sensitive gene expression analysis, and the properties of NGS data permit comparison between different studies. For further comparison, testis transcriptome data from an additional study was analyzed [36] (Table 1). To generate comparable expression data, we reanalyzed the human NGS transcriptome data starting from the fastq raw data sets.

**Table 1:** Summary of RNA-Seq data.

Sample	Organism	read length	read structure	Total prepared	Reads with at least one reported alignment (%)	Source
Adipose	human	75 bp	single	76269225	71283632 (93%)	Body Map 2.0
Adrenal	human	75 bp	single	76171569	71161611 (93%)	Body Map 2.0
Brain	human	75 bp	single	64313204	60789593 (95%)	Body Map 2.0
Breast	human	75 bp	single	77195260	71996826 (93%)	Body Map 2.0
Colon	human	75 bp	single	80257757	75646124 (94%)	Body Map 2.0
Heart	human	75 bp	single	76766862	72517553 (95%)	Body Map 2.0
Kidney	human	75 bp	single	79772393	74520114 (93%)	Body Map 2.0
Liver	human	75 bp	single	77453877	72420736 (94%)	Body Map 2.0
Lung	human	75 bp	single	81255438	75348205 (93%)	Body Map 2.0
Lymph Node	human	75 bp	single	81916460	74638341 (91%)	Body Map 2.0
Ovar	human	75 bp	single	81003052	76139023 (94%)	Body Map 2.0
Prostate	human	75 bp	single	83319902	78237991 (94%)	Body Map 2.0
Skeletal Muscle	human	75 bp	single	82864636	76882152 (93%)	Body Map 2.0
Testis	human	75 bp	single	82044319	76801169 (94%)	Body Map 2.0
Testis 2	human	32 bp	single	28654069	25767031 (90%)	Wang et al., 2008
Testis 3	human	50 bp	paired-end	81836199	left: 77403519 (95%)	Body Map 2.0
					right: 75427140 (92%)	
Thyroid	human	75 bp	single	80246657	74860275 (93%)	Body Map 2.0
Thyroid 2	human	50 bp	paired-end	81912887	left: 78512529 (96%)	Body Map 2.0
					right: 76452409 (93%)	
White Blood Cells	human	75 bp	single	82785673	77253598 (93%)	Body Map 2.0

Sequencing results were analyzed by TopHat and Cufflinks software. Reads were mapped onto the human reference genome (hg19). Expression values were calculated for each sample based on the number of fragments per kilobase of exon per million fragments mapped (FPKM) [37] (Table S1). In a rough scale, 1 FPKM corresponds to weak expression, 10 FPKM to moderate expression and 100 FPKM to high expression. As a basis for comparison, we calculated the FPKM values for typical housekeeping genes. For example, the strongly expressed ß-actin gene yields an expression value between ∼500–5000 FPKM, while the weakly to moderate expressed TATA box binding protein is detected at ∼1.6–21 FPKM (Figure S1). To gain an impression of FPKM values for the expression of genes, we calculated a histogram of FPKM value distribution for brain tissue (Figure S2). Our analysis detected the expression of ∼17000 genes in the appropriate tissue (FPKM >0.1 out of all ∼23000 genes).

We next defined a threshold for classification of a gene as expressed. A comparison of mapped reads between exon and intergenic regions was used to estimate an FPKM value that indicates with high confidence a level of gene expression above background level, as described by Ramsköld et al. [32] (Figure S3). We set the threshold value at 0.1 FPKM, which is slightly higher than the intersection of the false discovery rate and the false negative rate (Figure S3D). However, our statistical analysis showed that genes below this threshold with FPKM values between 0.01 and 0.1 are also truly positive in 83.5% of cases. We decided to leave these expressed ORs in our analysis, labeling them as a group of ‘potentially expressed’ genes. This was partially based on the fact that the analyzed RNA was derived from complex organs that contain different cell types; we supposed that small FPKM values between 0.01 and 0.1 could indicate the expression of genes in a small number of specific cells. In addition, we compared two independent sequencing runs of the same tissue to a different tissue. We detected 51 ORs in the 75-bp single-read data set of testis tissue within a range of 0.01–0.1 FPKM. Sixty-seven percent of these ORs were also detected in the independently sequenced paired-end testis data set, indicating that the most of the OR transcripts within this expression range are truly positive and not artificial. In contrast, we detected only 6% (3 of 51) of these ORs in the analysis of skeletal muscle data. This indicates that the weakly expressed ORs are not derived from randomly distributed mapped reads (Figure S4).

**Figure 1:**
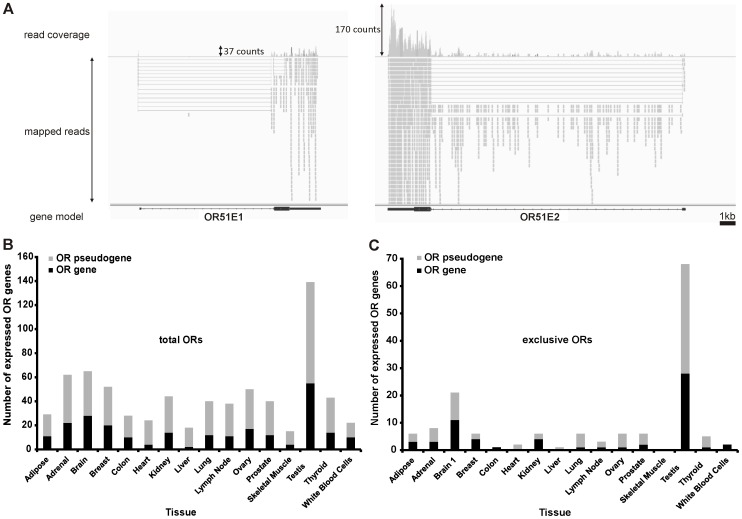
Detection of weakly and highly expressed ORs with RNA-Seq. A: Sample representation of read coverage of weakly and highly expressed ORs detected in the prostate and visualized by the Integrative Genomic Viewer. The gray segments indicate reads that were mapped onto the reference genome. The gene is indicated by black bars (exon) and thin lines (intron). Above, the read coverage is shown (detected and mapped counts/bases at each respective position). **B:** Each bar represents the number of OR genes (black) or OR pseudogenes (gray) that were expressed in one of the 16 investigated tissues with an FPKM value >0.1. The largest number of ORs were detected in testis, brain and ovary; only a few ORs were detected in skeletal muscle and liver. **C:** The bar diagram shows the number of ORs exclusively expressed in each tissue. Exclusively expressed ORs have greater FPKM values than 0.1 in the tissue indicated and are expressed at FPKM values lower than 0.1 in all other tissues. Testis had the greatest number of exclusively expressed ORs. In skeletal muscle, no exclusively expressed ORs were detected.

We analyzed the expression of ORs in each of the 16 different tissues. Due to the number of generated sequences in the Body Map project 2.0, the expression of ORs is typically confirmed by hundreds to thousands of mapped reads. For example, the highly expressed OR51E2 gene (8.5 FPKM in prostate) is confirmed by 2320 mapped reads, and the less highly expressed OR51E1 gene (0.8 FPKM in prostate) is confirmed by 353 reads (Figure 1A). Calculated for prostate tissue with 78 million aligned reads, an FPKM value of ∼0.01–0.02 represents two mapped reads in a 1-kb exon.

### Expression of OR Genes

We detected ectopically expressed OR genes in each of the 16 tissues investigated. The lowest number of expressed OR genes was found in liver (2 ORs), and the highest number was found in testis (55 ORs) (Figure 1B; FPKM >0.1). In each tissue except skeletal muscle, some ORs were exclusively expressed (Figure 1C). We calculated the cumulative expression of ORs (the sum of all OR FPKM values per tissue) and found that OR genes are more highly expressed in testis than in any other tissue (Figure S5). We checked the sequence similarities of the most highly ectopically expressed OR genes. Due to their coding sequence similarity (99–100%), the FPKM values of OR2A4 and OR2A7, as well as those of OR2A1 and OR2A42 are presented together.

**Figure 2:**
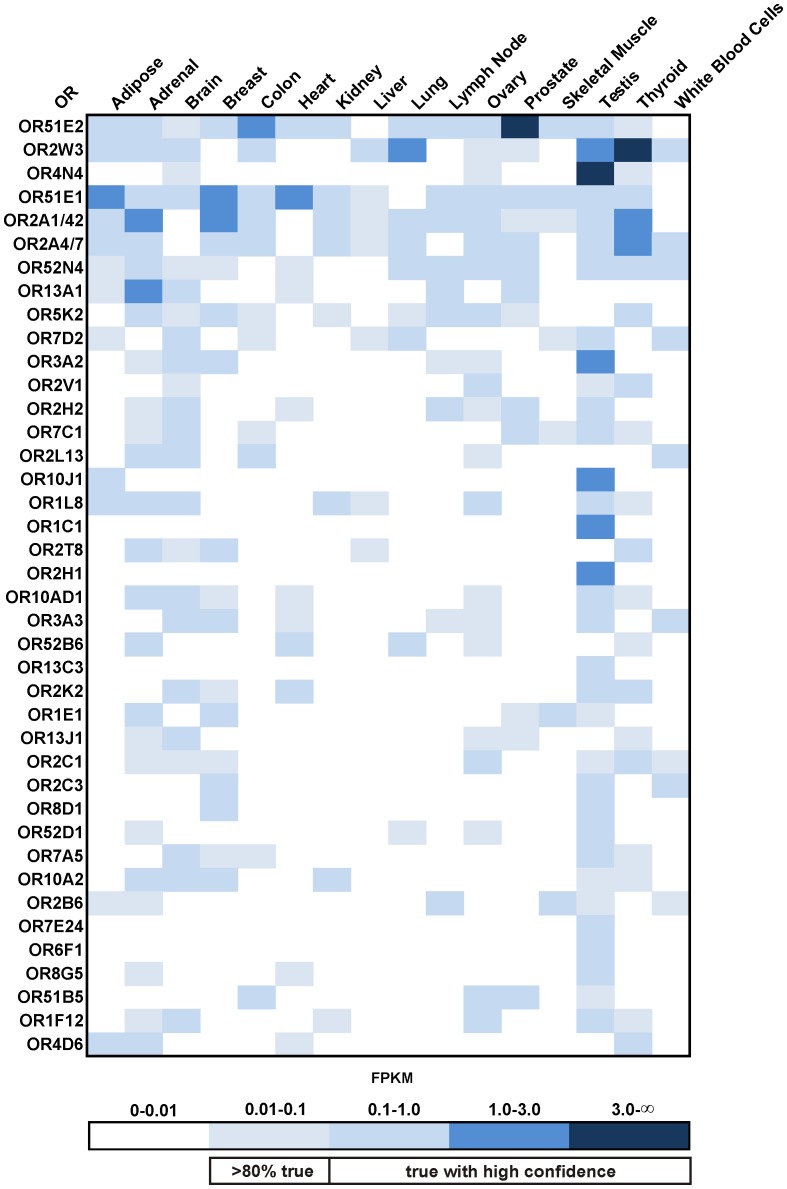
Expression pattern of ectopically expressed ORs. The heat map shows FPKM values for the 40 most highly expressed ORs found in the human tissues studied. Dark blue indicates high expression (FPKM values higher than 3), and white indicates no expression. ORs are sorted by the sum of their expression values across all tissues.

We next analyzed OR gene expression patterns. Of the 400 OR genes, 111 ORs showed FPKM values higher than 0.1, and 10 showed values higher than 1 FPKM, in at least one tissue. Ninety-one additional ORs were expressed at an FPKM between 0.01–0.1 in at least one tissue; these were regarded as potentially expressed genes (Figure S3). The three most highly expressed receptors were OR4N4 in testis (9.9 FPKM), OR51E2 in prostate (8.5 FPKM) and OR2W3 in thyroid (5.1 FPKM). To establish a general ranking of ectopically expressed ORs, we calculated the sum of all FPKM values for each respective OR in all 16 tissues. In total, 40 ORs had cumulative FPKM values of at least 0.5; these were defined as the most highly ectopically expressed ORs. Interestingly, we observed that a wide variety of tissues expressed similar sets of ORs. Of the broadly expressed ORs, OR51E2 was found in 12 different tissue samples, OR51E1 was found in 13 samples, and OR2W3 was found in 9, while several other specific transcripts were found in 13 or fewer tissues (FPKM >0.1; Figure 2). As expected, we detected high expression of OR51E2 in prostate, but it was also expressed in various other tissues. Other widely expressed receptors were OR52N4 and OR2A4/7. Several receptors were specifically expressed in only one tissue, primarily in testis (OR4N4, OR6F1 and OR2H1) (Figure 1C and 2). No specific ORs were found in skeletal muscle.

### Expression of OR Pseudogenes

**Figure 3:**
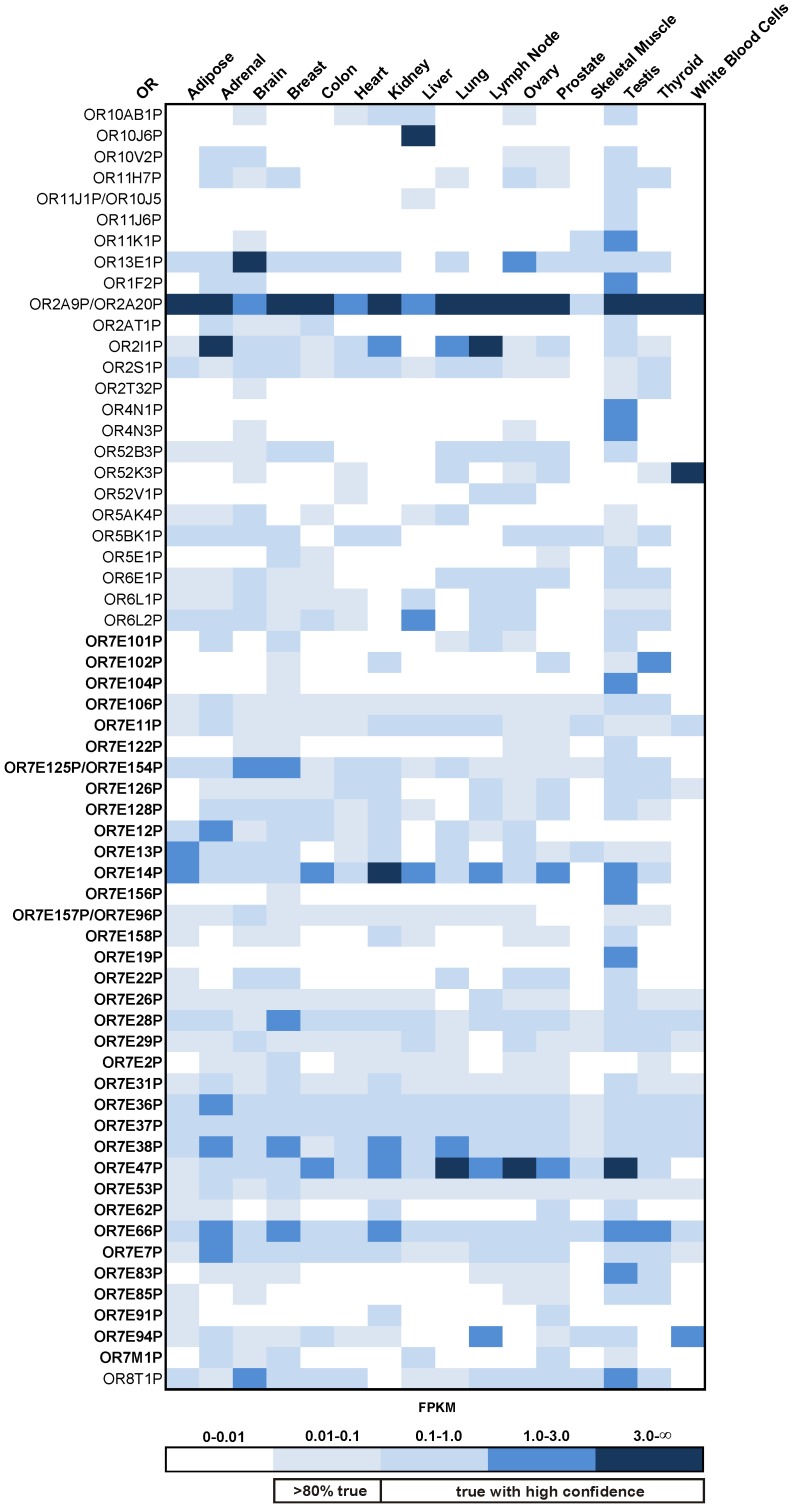
Pattern of expression of OR pseudogenes in different tissues. All OR pseudogenes with summed FPKM values >0.5 across all 16 from Body Map 2.0 are listed. The color intensity represents the FPKM value. Of the expressed pseudogenes, 58% belong to the 7E subfamily; genes in this family are indicated by bold letters.

There are slightly more annotated OR pseudogenes than OR genes; in contrast to OR genes, OR pseudogenes lack full open reading frames (ORF). In our analysis, some OR pseudogenes showed a broad expression pattern. We detected expression of 254 of these OR pseudogenes in at least one tissue (FPKM >0.01). Of these, 144 were detected in at least one tissue with an FPKM value higher than 0.1. In the 16 tissues, we detected 62 OR pseudogenes with summed FPKM values higher than 0.5; of these, 58% belong to the OR7E subfamily, the largest subfamily in the human OR repertoire (Figure 3). This subfamily has most likely expanded in the human genome through a series of segmental gene duplication events [38]. OR7E24 is the only member of this family that is not a pseudogene. In general, OR pseudogenes showed FPKM values similar to those of the OR genes. In some cases, OR pseudogene expression exceeded the expression of OR genes. For example, OR7E47P showed an FPKM value of 24 in lung tissue, and OR2A9P and OR2A20P were nearly ubiquitously and highly expressed, with FPKMs of up to 20. Other highly but more specifically expressed OR pseudogenes were also found, for example, OR10J6P in liver (5.3 FPKM), OR52K3P in white blood cells (5.3 FPKM), and OR7E19P (2.6 FPKM) and OR4N1P (2.2 FPKM) in testis (Figure 3). We checked the sequence similarities of the most highly ectopically expressed OR pseudogenes. The OR pseudogenes that showed sequence similarities between 99 and 100% are listed together (Figure 3).

### ORs in Testis

**Figure 4:**
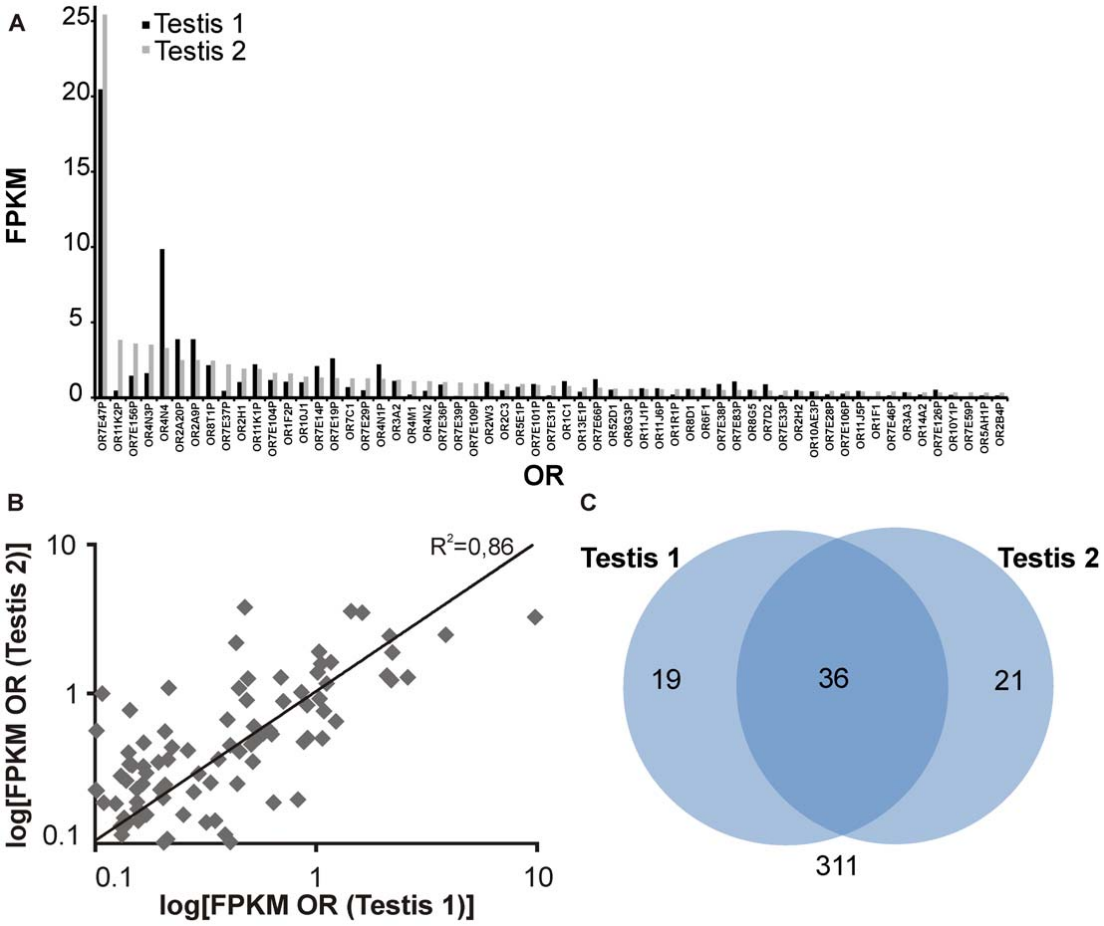
Expression of ORs in testis. A: Expression profile for the 60 most highly expressed OR genes and pseudogenes in two testis samples, sorted by FPKM values of Testis 2. **B:** Plotted expression pattern correlation for all detected ORs in two testis samples. R^2^ is the coefficient of determination. **C:** Venn diagram showing the intersection of OR transcripts detected in two independent RNA-Seqs of human testis (FPKM >0.1). In both RNA-Seq analyses, we detected 36 identical ORs. (Testis 1 = Body Map 2.0; Testis 2 = Wang et al., 2008).

The expression of ORs in testis has been reported for a number of species [13], [39], [40]. Our study identified testis as the tissue with the largest number of detected OR transcripts (Figure 1 and 4A). Comparison of the two RNA-Seq data sets for testis used in this study (Table 1) showed that 36 ORs were detected in testis using either data set (Figure 4C). The high expression of OR4N4 found in both RNA-Seq data sets is striking (Figure 4B). While human OR1D2 could be detected in both data sets, it showed lower FPKM values than the most highly expressed ORs and was not exclusively expressed in testis, confirming the results of Feldmesser and colleagues [28]. Several studies have reported an expression of the MHC-linked ORs, which are localized within a cluster on the short arm of chromosome 6 [9], [41]. In total, 6 of the 15 MHC-linked OR-genes were detected in both investigated testis samples. OR2H1, a member of this prominent group, is testis-specific.

### OR Expression Analysis

To identify potential artificial results that might be caused by nearby or overlapping highly expressed genes, we analyzed the distribution of mapped reads for the 20 most highly expressed OR genes in all tissues using the Integrative Genomic Viewer. The possible interference of adjacent highly expressed genes was suggested previously [28]. In contrast to microarray or RT-PCR data, RNA-Seq permits detailed analysis at the level of *de novo* detection of exons and splice sites. For highly expressed OR genes such as OR51E1 and OR51E2, the aligned sequences in known exon regions had equal distributions, while intron-spanning reads were found to connect known 5′UTRs with the ORF-containing exons (Figure 1A). No mapped reads were found within 2 kb up- or downstream of the adjacent OR gene regions.

**Figure 5:**
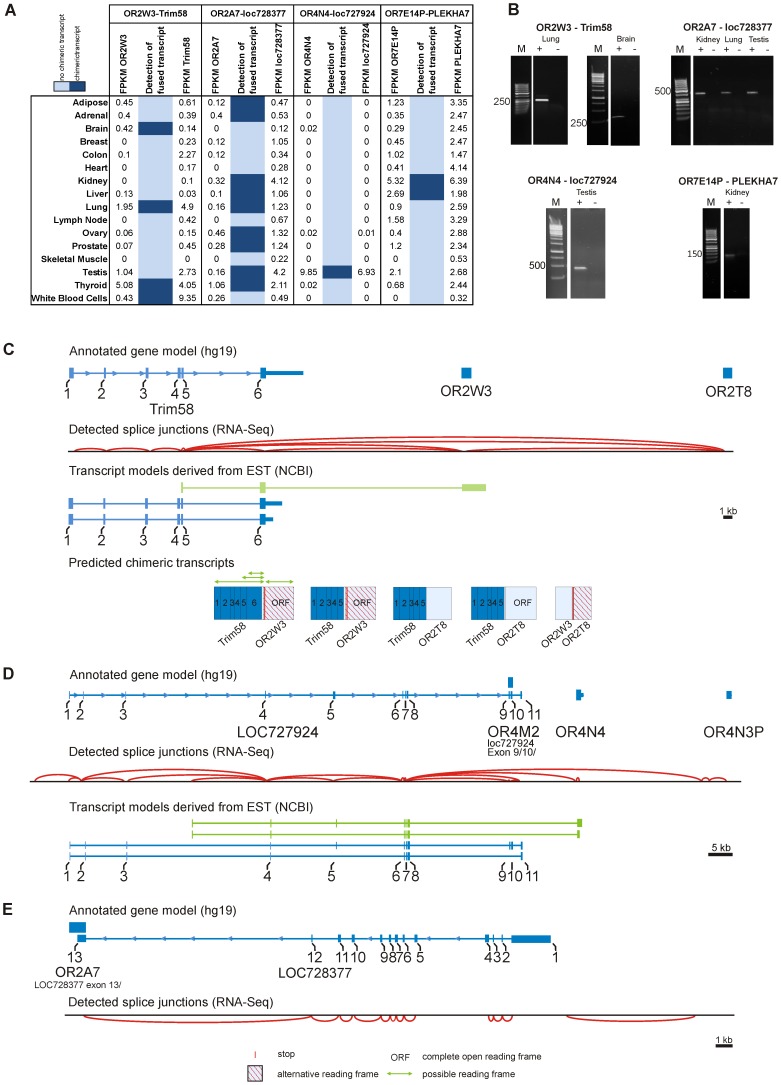
Chimeric transcripts of different ectopically expressed ORs in various tissues . **A:** The table shows expression values for four different ORs and their respective nearby genes in various tissues. Corresponding chimeric transcripts could be detected in different tissues. **B:** PCR experiments confirmed the observed chimeric transcripts of these ORs. OR2W3 shares chimeric transcripts with the Trim58 gene, while OR2A7 shares chimeric transcripts with part of loc728377. In testis only, OR4N4 shows chimeric transcripts with the loc727924 gene. The OR pseudogene OR7E14P yield chimeric transcripts with the Plekha7 gene. We confirmed the amplified PCR products by Sanger sequencing. **C:** Schematic representation of the detected chimeric transcripts of Trim58 with OR2W3 and OR2T8. RNA-Seq of thyroid tissue reveals a complex splice pattern (red arcs) leading to chimeric transcripts of Trim58 and OR2W3 or OR2T8 as well as chimeric transcripts of OR2W3 with OR2T8. The green arrows indicate ORFs. Depending on the used splice sites, the reading frame of the odorant receptor can, in principle, be intact or fused to the Trim58 reading frame. **D:** Splicing between the uncharacterized loc727924 gene and the OR4N4 or OR4N3P in testis. Parts of the coding exon of OR4M2 gene overlap with exons of the loc727924 gene. **E:** Chimeric transcripts of OR2A7 and loc728377 in kidney. The coding exon of OR2A7 overlaps with exon 13 of the loc728377 gene.

The expression pattern of other ORs with high FPKM values or broad expression was more complex (Figure 5). In several tissues, the FPKM values of OR2W3 and the nearby gene Trim58 correlate with each other (Figure 5A). According to our splice analysis, OR2W3 shares exons with Trim58 (Figure 5C). RNA-Seq indicated the presence of chimeric transcripts that code for a prematurely terminated Trim58 protein but also contain the complete intact coding sequence of OR2W3; however, the OR2W3 coding sequence is not in frame with the Trim58 coding sequence (Figure 5C). The detected chimeric transcripts were confirmed by RT-PCR with a forward primer located in exon 5 of the Trim58 gene and a reverse primer located in the ORF of OR2W3 (Figure 5B). The analysis of available EST clones confirmed the presence of chimeric transcripts of Trim58 and OR2W3. We found clones containing only the last two exons, 5 and 6, of Trim58, indicating a potential different initiation site of transcription (Figure 5C). However, RT-PCR experiments with a forward primer located in exon 3 or 4 of Trim58 and a reverse primer located in the ORF of OR2W3 revealed a specific fragment, indicating that the EST clones may be incomplete and that further upstream exons belong to the chimeric transcript (Figure S7). Therefore, our results suggest that OR2W3 and Trim58 expression are under control of the same promoter. In this case, it seems that OR2W3 expression is a byproduct of erroneous splicing. Nevertheless, in some human tissues, the expression values of OR2W3 exceed those of Trim58 (Figure 5A). We were not able to detect chimeric transcripts in every tissue by RNA-Seq; this indicates different control of the expression of OR2W3 in some tissues. We also detected splice junctions from exon 5 of Trim58 to OR2T8. These transcripts code for a part of Trim58 and the complete intact coding sequence of OR2T8 (Figure 5C). Translation of this protein could encode a new protein combining features of Trim58 and OR2T8. Similar shared 5′UTR exons have been demonstrated for OR5V1 and OR12D3 within the MHC locus [9]. A similar constellation of fused transcripts was observed in the paired-end data sets for thyroid tissue (data not shown).

A slightly different situation was found for OR4N4 expression. The OR4N4 gene has chimeric transcripts with an uncharacterized non-coding RNA loc727924. Both are almost exclusively present in testis (Figure 5A and D). Chimeric transcript expression was verified by RT-PCR (Figure 5B). Both EST clones and RNA-Seq of loc727924 and OR4N4 revealed a complex expression pattern in this gene region. EST clone analysis confirmed the presence of the chimeric transcripts loc727924 and OR4N4. All of these transcripts start from an unannotated 5′UTR exon located between exons 3 and 4 of loc727924. Consistent with this, we detected splice junctions from this unannotated exon to the annotated exon 4 of loc727924 but no splice junctions derived from the upstream exons of loc727924, indicating independent expression of OR4N4 (Figure 5D). These findings suggest that two types of transcripts are expressed. One variant starts at exon 1 of loc727924 but never contains OR4N4. The second variant starts with a 5′UTR consisting of the newly identified exon between exons 3 and 4 of loc727924 and exons 4–8 of loc727924 followed by the complete coding sequence of OR4N4. Fused transcripts of loc727924 and OR4N3P could also be detected.

In two further RT-PCR verified cases, expression may have been partly caused by or influenced by nearby genes. First, OR7E14P shares chimeric transcripts with the Plekha7 gene. Second, a part of the OR2A7 ORF overlays with exon 13 of loc728377 (Figure 5B and E). Because these genes overlap, it is difficult to speculate whether OR2A7 or the non-coding loc728377 is expressed.

**Figure 6:**
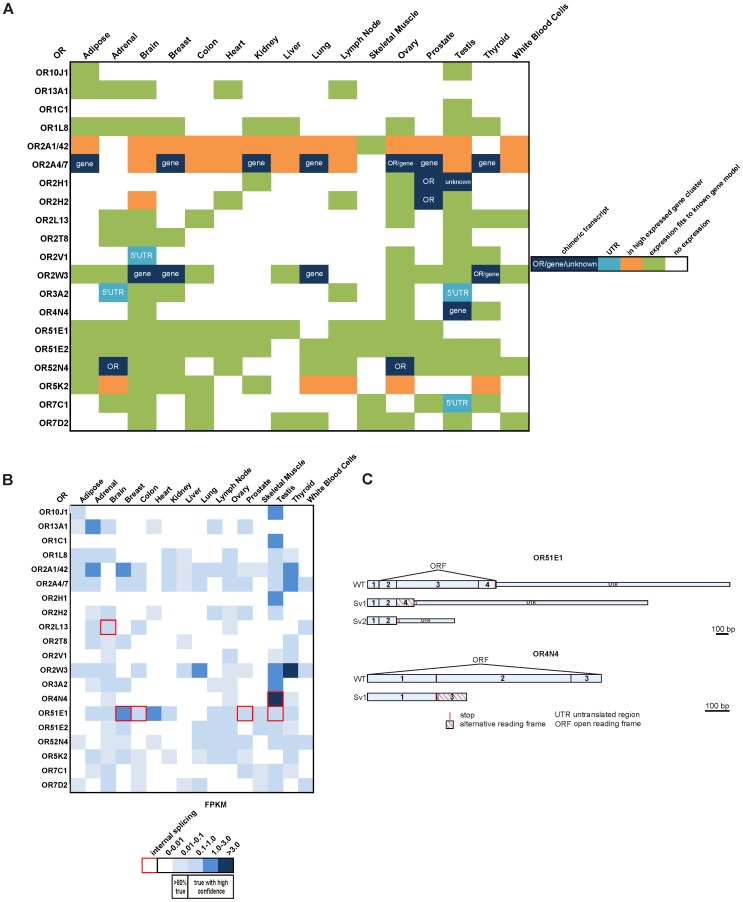
Analysis of OR transcripts across tissues. A: Analysis of the 20 most highly expressed ORs (summed FPKM >1). The graphic illustrates the presence of detected chimeric transcripts or unannotated untranslated regions in the upstream areas of the respective OR ORF. **B:** Overview of detected internal splicing events within the ORF of the 20 most highly expressed ORs. The heat map indicates the level of expression of the respective receptor and the detected internal splicing events (red frames). **C:** Schematic representation of detected internal splicing events of the broadly expressed OR51E1 and the testis-specific OR4N4.

We analyzed the 20 most highly expressed ORs and detected 6 ORs that form chimeric transcripts with upstream genes in at least one investigated tissue. In these cases, expression might be caused by nearby genes or involve unknown 5′UTRs that lie within nearby genes (Figure 6A). In some cases, no annotated genes could be located in the upstream OR regions, indicating common 5′UTRs for many OR transcripts.

For the 20 most highly ectopically expressed ORs, we also checked for splicing events within the ORF (Figure 6B). In some tissues, two different internal splice variants of OR51E1 were observed. OR4N4, which almost exclusively occurs in testis, has one internal splice variant (Figure 6C). The occurrence of splicing events similar to these has been described in recent studies [8], [9]. In consequence, the fraction of mRNAs with intact ORFs is reduced. Furthermore, we observed that some genomic regions are highly and uniformly expressed. OR expression could not be specifically assigned when ORs are located in such a cluster. For example, OR13E1P in brain is located in a highly expressed gene cluster of ∼10 kb (Figure S8). We also focused on the dependence of OR expression on the genomic environment to investigate whether the expression of non-OR gene neighbors influences the expression of ORs (Figure S9). We found that 71% of expressed ORs and OR pseudogenes were located next to at least one non-OR gene. In contrast, 43% of ORs and OR pseudogenes that had an OR neighbor were ectopically expressed in at least one tissue. The results indicate that ORs with a non-OR neighbor have a higher tendency to be expressed than ORs that are located next to other OR genes.

### Verification of OR Expression Results by RT-PCR

**Figure 7:**
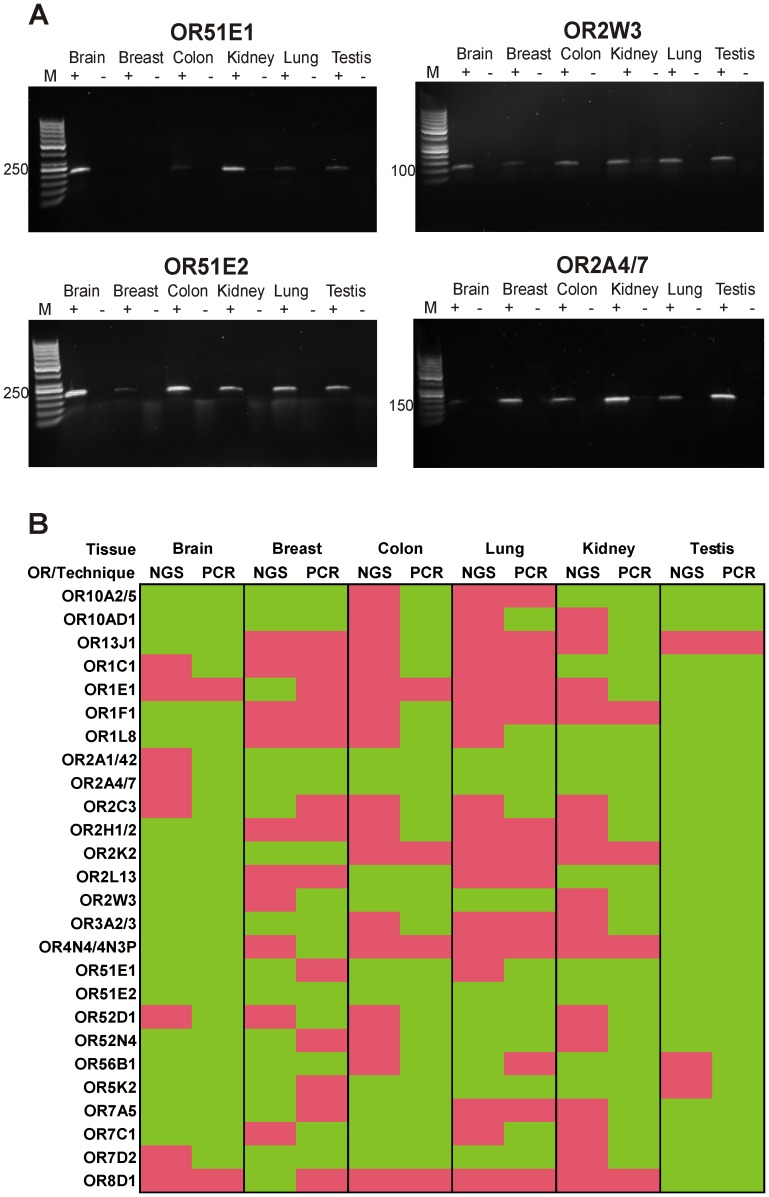
Validation of RNA-Seq results using RT-PCR. A: Gel electrophoresis of the amplified PCR products from cDNA samples (+) of brain, breast, colon, kidney, lung and testis. The RNA of the investigated tissues does not contain genomic DNA contamination, as shown in (**−**). The presence of broadly expressed ORs detected by RNA-Seq could be confirmed by RT-PCR. **B:** Table showing the summarized PCR validation in comparison to RNA-Seq data. We investigated 26 different ORs that showed broad or high expression. Green color indicates the detection of ORs with the respective technique; red color indicates no detection.

Today, RT-PCR is considered the “gold standard” for expression analysis. In this study, we used RT-PCR to confirm several examples of the NGS expression data from six tissues. Because we do not have access to the original samples analyzed by the Body Map project, we used commercially available RNA samples from human brain, breast, colon, kidney, lung and testis. Although these tissues are from other donors than those who provided tissues for the Body Map project, we could validate the expression of the interesting broadly expressed ORs (Figure 7A). For four of the six investigated tissues we confirmed all of the NGS-detected ORs by RT-PCR. One OR in lung tissue and seven of 26 ORs in breast tissue could not be confirmed by PCR (Figures 7B and S10). In some cases, we detected expressed OR genes by RT-PCR but not by NGS sequencing, indicating that RT-PCR is more sensitive than our RNA-Seq analysis for extremely weakly expressed transcripts. Additionally, we cannot exclude differences between tissues obtained from different donors. In this context, our RT-PCR experiments indicate the expression of OR2W3 in breast and kidney, whereas the NGS data did not. We found that OR51E1 was not detected with PCR in our available breast tissue despite a robust FPKM value for this gene in the RNA-Seq analysis. OR51E1 was detected in the lung RNA sample by RT-PCR. OR2A4/7, which is not detectable in brain RNA by deep sequencing, was evident by RT-PCR in the brain RNA sample. Despite the use of RNA samples from different donors in the RT-PCR and NGS sequencing experiments, it was in general possible to confirm broad and exclusively expressed ORs by RT-PCR. Furthermore, our experiments indicated that there might be differences in OR expression between different donors, depending on the exact localization of the tissue samples and the gender and age of the donors.

### Signaling Pathway Components and Other Chemosensors

Next, we determined the expression pattern of components other than ORs that are involved in olfactory signal transduction. Data on the expression of such components can serve as a hint for the existence of downstream olfactory signal transduction. As an example, Pluznick et al. measured the expression of key components of olfaction (ORs, adenylyl cyclase III and the Gα subunit Gα_olf_) in the kidneys of mice and suggested a functional role for these components in the modulation of renin secretion and glomerular filtration rate [27].

**Figure 8:**
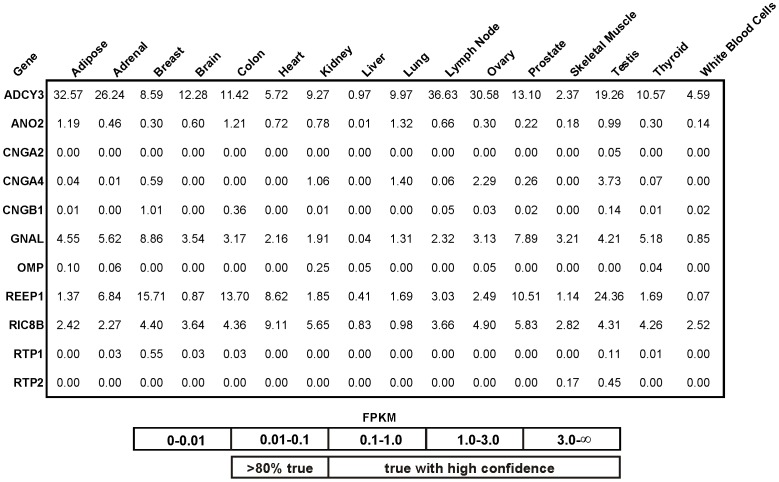
Expression of signaling pathway components across tissues. Expression analysis of signaling components including, Gα_olf_ (GNAL), adenylyl cyclase III (ADCY3), CNG channel subunits (CNGA2, CNGA4 and CNGB1), calcium-activated chloride channel (ANO2) and the nucleotide exchange factor Ric8b (RIC8B). We also investigated the expression of accessory proteins including receptor-transporting proteins (RTP1 and RTP2) and receptor-enhancing proteins 1 (REEP1), as well as the expression of the olfactory marker protein (OMP), a specific marker for olfactory sensory neurons.

In olfactory neurons, binding of an odorant to its appropriate OR leads, in principle, to the activation of a cAMP-activated second messenger pathway that involves Gα_olf_, adenylyl cyclase III and the CNG channel subunits CNGA2, CNGA4 and CNGB1 [42]. Gα_olf_ signaling is enhanced by the nucleotide exchange factor Ric8b [43]. Our analysis indicates that the basic components of olfactory signal transduction components, the specific Gα subunit Gα_olf_ and the adenylyl cyclase III, could be detected in all tissues. However, the CNGA2 subunit of CNG channels was only detected in testis (Figure 8).

**Figure 9:**
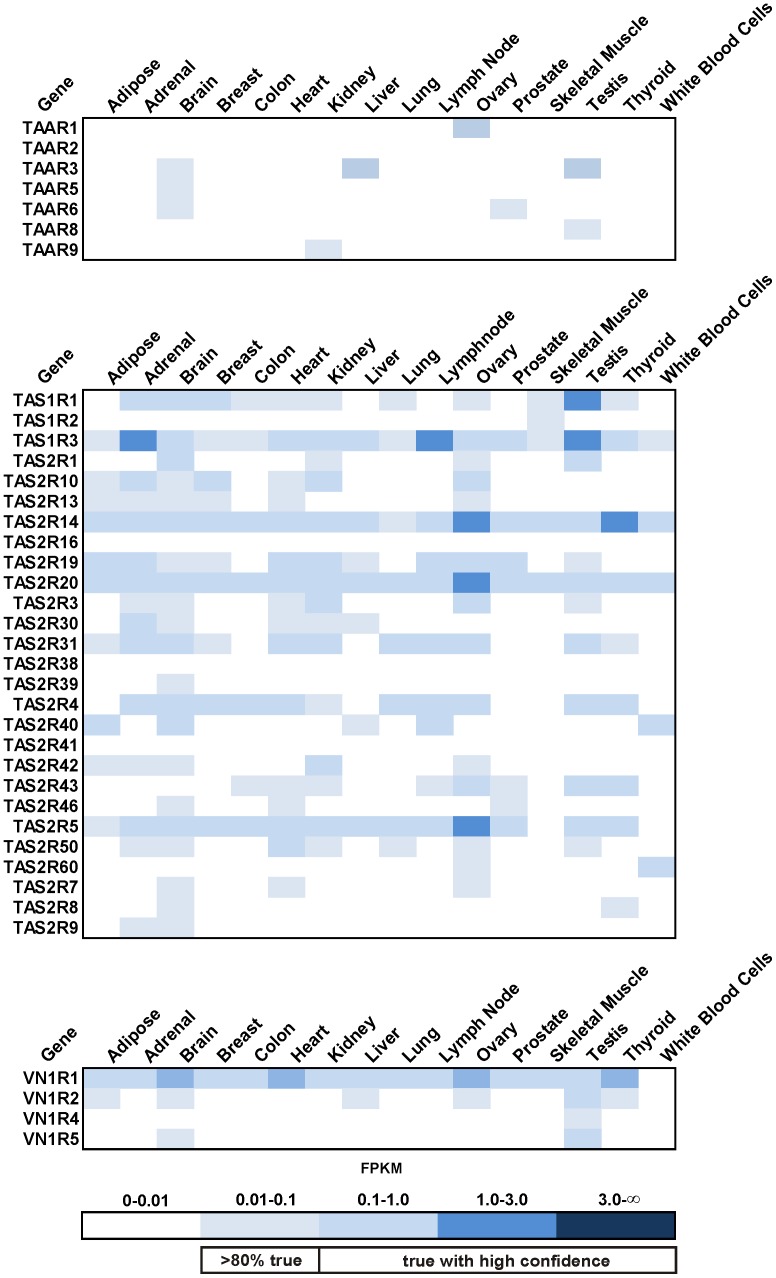
Expression of other chemosensors in human tissues. TAARs show very weak or no expression in the investigated tissues, while the taste receptors TAS1R and TAS2R show detectable expression across the investigated tissues. The vomeronasal receptors (VN1R), namely VN1R1, show a widespread expression pattern.

In the last step of our analysis, we focused on the expression of other GPCR-type chemoreceptors, namely trace amine receptors (TAARs), bitter (TAS2Rs) and sweet/umami receptors (TAS1Rs) and vomeronasal type 1 receptor (VN1R). VN1R1 was detected in various tissues by RNA-Seq (Figure 9). The taste receptors TAS1R3 (sweet taste receptor), TAS2R14 and TAS2R20 (both bitter taste receptors) showed broad expression in all 16 investigated tissues. While TAAR1 expression in ovary is appreciable, the expression patterns for other TAARs are weak.

## Discussion

Comprehensive analyses of the ectopic expression of ORs in human tissues are still rare. In this paper, we analyzed transcriptome data generated by the relatively new NGS method RNA-Seq. This method allowed a comparable and quantitative investigation of OR expression at the mRNA level. Previous studies have investigated OR gene expression by microarray analysis, which has methodical limitations for the analysis of ectopically expressed ORs [29], [44]. Using the RNA-Seq approach, we were able to determine the expression of OR genes and pseudogenes in various tissues independently, in contrast to microarray analysis, which only allows the comparison of OR expression levels between different tissue samples [44]. RNA-Seq has been shown to be highly accurate for quantifying expression levels, and its accuracy has been confirmed many times by quantitative PCR [45]. Weakly expressed genes that are difficult to analyze with microarrays can be detected by NGS at a high sequencing depth (>40 million reads) [37]. With more than 70 million generated reads for each tissue in the Body Map project 2.0, it is obvious that these data provide a more accurate basis for comparison of gene expression than microarray analysis [46]. We established a comprehensive analysis of ectopically expressed human ORs using the suitable method of RNA-Seq; the results of this analysis represent an important step towards understanding the molecular basis of ectopically expressed ORs.

### Ectopically Expressed ORs

In general, we found that 111 of the 400 ORs in the genome are expressed with high confidence in at least one tissue (FPKM >0.1). We detected transcripts of different ORs in all 16 of the human tissues we investigated. However, the number of expressed OR genes was tissue-dependent; it ranged from 2 in liver to 55 in testis. This indicates considerably lower OR expression than was previously estimated based on microarray data from human olfactory epithelium (76% of all ORs) [29] or RNA-Seq data of murine olfactory epithelium, in which virtually all ORs are expressed [47].

In agreement with previous studies, our results indicate broad ectopic expression of some ORs [28], [29], [44], [48]. Previous studies have indicated that lung and heart display the highest number of ectopically expressed ORs [28], [29]. Our data, however, clearly identify testis as the tissue with the highest number of expressed ORs, and indicate a possible important functional role of ORs in testis. However, the functionalities of the majority of ectopically expressed ORs are uncertain because functional data are still sparse. The majority of the most highly ectopically expressed ORs (73%) are also expressed in the human olfactory epithelium (Figure S12). We suggest that ectopically expressed ORs do not form a separate group with other functions than ORs that are expressed in the olfactory epithelium.

A large number of ORs are pseudogenes. In our study, 31% of all OR pseudogenes were expressed in at least one non-olfactory tissue. Previous studies have described pseudogene expression in olfactory epithelium. It was hypothesized that a suggested nonsense-mediated decay RNA system might not remove OR pseudogene mRNA and that the expression of OR pseudogenes may play a role in the regulation of OR gene expression in olfactory sensory neurons [29]. Expression of OR pseudogenes in non-olfactory tissues may be involved in similar processes of gene regulation, but the exact reason for the expression of OR pseudogenes is unclear.

### Expression Level of ORs

Based on our calculated FPKM values, mRNA for most ORs is of low abundance. In an expression ranking of ∼23000 genes, ORs are typically found at positions >10000. Overall, we detected the expression of 111 ORs at FPKM higher than 0.1 in at least one tissue. Compared to housekeeping genes, the FPKM values of ORs indicate weaker gene expression; their level of expression is roughly comparable to that of the TATA box binding protein, with an average expression value of 3 in the case of OR51E2. In many cases, the FPKM values of OR genes were lower than 1, indicating that their overall expression in the respective tissues is weak.

The RNA samples investigated in this study were all extracted from whole organs. Therefore, it is possible that ORs are highly expressed in specific cell types that represent a small fraction of the tissues that make up these organs. A similar pattern, referred to as mosaic gene expression, could be shown for TAAR1 in brain [49]. In several reports describing the ectopic expression of ORs, it was shown that only a few cells in some complex tissues express ORs, adenylyl cyclase III or Gα_olf_. For instance, in the gut, ORs were detected in human gastrointestinal enterochromaffin cells, which constitute only a minor proportion of the total intestinal epithelium and have a diffuse distribution [18]. In mouse kidney, Gα_olf_ and adenylyl cyclase III were detected at the RNA and protein levels in the distal nephrons [27]. In RNA isolated from these tissues, RNA from these cells is extremely diluted; thus, the respective FPKM values are low or expression is not detectable. For similar reasons, we cannot assess whether the low FPKM values observed in our study depend on generally low expression or on mosaic expression. We suggest that expression of ORs even at very low FPKM values could be meaningful and could indicate the involvement of these gene products in physiological processes.

The investigation of ORs at the cellular resolution level by *in situ* hybridization or immunohistochemistry of whole tissue slices might be a suitable approach to localize the expression of OR transcripts or proteins and would indicate whether there are specialized cell types or areas that highly express OR transcripts. In this regard, it is plausible that there might be other expressed ORs in different tissues that are not detectable by RNA-Seq analysis of whole organs or complete tissues. The detection of ORs at the protein level is important to gain more information about the functionality of ectopically expressed ORs.

Our RT-PCR validation confirmed the NGS data. We measured the expression of 26 OR genes in six different tissues (brain, breast, colon, kidney, lung, testis) by RT-PCR. We selected ORs that showed broad expression or high expression in specific tissues. In five of the six investigated tissues, all receptors detected by RNA-Seq, with the exception of only one receptor in lung tissue, were confirmed by RT-PCR. For breast tissue, we confirmed only 56% of the NGS-detected ORs by RT-PCR. The breast tissue investigated by RNA-Seq was obtained from a 29-year-old female, whereas the breast tissue used for our RT-PCR experiments originated from a 52-year-old female. The expression pattern of ORs in breast tissue might change with age or as a result of environmental influences. Although the cDNA probes used for RT-PCR were prepared from different tissue samples than the Body Map project tissues, the OR expression pattern in five of the six investigated tissues was conserved. Furthermore, we detected more ORs by RT-PCR than by RNA-Seq. Discrepancies between RT-PCR and RNA-Seq could be expected due to the higher sensitivity of RT-PCR in comparison to our RNA-Seq data sets. The number of ORs detected by RNA-Seq may therefore be regarded as the minimum number of expressed ORs in the respective tissue.

### Most Highly Ectopically Expressed ORs

Our analysis provides a list of OR genes that are transcribed in a variety of human tissues. To the best of our knowledge, ligands for only two of the 20 most highly ectopically expressed ORs, are known [17], [50], [51]. The ß-ionone receptor OR51E2 is highly expressed in prostate tissue. The prominent expression of OR51E2 and its possible physiological functions have already been described [17], [52]. The second deorphanized OR is the 3-methyl-valeric acid and nonanoic acid receptor OR51E1, which was recently postulated as a marker for neuroendocrinic carcinoma cells and is overexpressed in human prostate cancer [53], [54]. Fujita and colleagues demonstrated that OR51E1 is also expressed in various human tissues, a finding that is consistent with our results [50]. Our investigations showed that there is almost ubiquitous expression of both these receptors in the tissues investigated in this study. The results suggest a general involvement of OR51E1 and OR51E2 in physiological processes, and the effect of their ligands on these tissues should be tested in the future. The other highly expressed orphan ORs are interesting candidates for further deorphanization studies, however, their functionality in a recombinant system has not yet been shown.

We analyzed EST and microarray data for five most highly ectopically expressed ORs which were available on NCBI. Ectopic OR2W3 expression is confirmed by EST data from fetal brain, blood and liver as well as with microarray data in breast cancer cells [55]. The RNA-Seq data showed that OR4N4 is highly and specifically expressed in testis, a finding that is confirmed by EST data. The expression of OR2A1 is known to be regulated in response to chemotherapy in ovarian cancer [56]. EST data indicate expression OR2A1 in lung and several tumors. As we have shown here, OR2A1/42 is broadly expressed in various human tissues. Consistent with our results, the EST data for OR2A7 indicate that it is expressed in more than 20 different human tissues. In general, RNA-Seq results for broadly expressed ORs are confirmed by EST data, and ORs with many known EST clones have higher FPKM values in our analyses. All of the available previous data indicate that there is broad expression of ORs in different tissues. Our data extend previous results and permit quantitative and comprehensive analysis of the expression of ORs in different tissues.

We found the largest number of OR transcripts in testis (55 ORs; FPKM >0.1); six of these ORs showed expression values higher than 1 FPKM. We also detected the well-characterized OR1D2 and OR7A5, as well as 40% of the MHC-linked ORs, in testis [11], [14]. It is speculated that the latter ORs may be involved in the detection of MHC peptides in testis and spermatozoa [41]. A number of studies have already addressed the expression and function of ORs in spermatozoa and testis of various species [10]–[14]. In addition to involvement in the process of chemotaxis in spermatozoa, ORs could be involved in sperm development and competition or interaction between spermatozoa and oocytes [39], [41], [57]. Our results support the idea that ORs are involved in physiological processes in testis and spermatozoa.

### Interference of OR Expression with Upstream Non-OR Genes

The use of RNA-Seq permitted expression analysis not only of all ORs as well as OR pseudogenes but also analysis of all other genes expressed in each tissue. With this advantage, we were able to investigate the possible interference of neighboring genes with OR gene expression. Feldmesser et al. reported that ORs located within 0.5 M of non-OR genes have a higher tendency to be expressed than others [28]. We confirmed this and also found that OR genes located adjacent to non-OR genes, have a higher tendency to be expressed. In addition, we detected chimeric transcripts consisting of ORs and upstream non-olfactory genes. Previous studies have reported the presence of chimeric transcripts for non-OR genes in the human Body Map 2.0 data sets from healthy tissues [58]. It is possible that the chimeric transcripts found in healthy tissues encode new proteins with different functions [59], [60]. In our study, we described chimeric transcripts for ORs and upstream genes and verified several by RT-PCR. The occurrence of such chimeric transcripts may explain the ectopic expression of some ORs in healthy non-olfactory tissues; the exact mechanisms and reasons for their production remain be discovered.

We also identified new, unannotated 5′UTRs for several ORs; these may represent chimeric transcripts shared with unannotated genes or a complex pattern of the 5′UTRs of ORs. A previous study showed that testicular OR gene transcripts are generated by a highly unorthodox combination of complex transcriptional events, including long-distance and intracoding exon splicing [9].

Another possible explanation of seemingly broad ectopic expression was suggested, for example, in the cases of OR5K2 and OR13E1P. These genes are located within a large and highly expressed gene region that is uniformly covered with aligned reads with no visible intron/exon structure. It is probable that aligned reads do not originate from any OR gene expression. Such artifacts in gene expression cannot be detected by RT-PCR or microarray analysis; however, they become obvious following detailed RNA-Seq analysis.

### Chimeric Transcripts with Trim58

We observed that in some tissues expression of OR2W3 correlates with the expression of Trim58. We found that OR2W3 shares transcripts with Trim58. The 5′ portion of the shared transcripts is coded by Trim58 (up to exon 6); it is spliced at its 3′ end to an exon containing the complete OR2W3 ORF. Due to the prediction that in most eukaryotes mRNA translation initiates at the first AUG starting from the 5′-cap [61], this transcript would code for a truncated Trim58 protein and not for a fused protein that also contains OR2W3. However, OR2W3 could be translated if the internal start ATG of the OR2W3 ORF were used. All trim genes (tripartite motif-containing genes) provide proteins that have three specific motifs (a RING finger, a B-box and coiled coil motifs). Trim proteins bind unwanted proteins and tag them with ubiquitin. They are involved in a broad range of biological processes, and some function as important regulators in carcinogenesis [62]. Interestingly, a previous study showed that mRNA expression for OR2W3 and Trim58 in blood cells is downregulated if beta-adrenergic receptor antagonists are applied [63].

OR2T8 is also part of a chimeric transcript with Trim58 that was detected in some tissues. In this case, the chimeric transcript codes for a chimeric protein comprising a portion of the N-terminus of the Trim58 protein and a portion of the C-terminus of the OR2T8 protein. Frenkel-Morgenstern and colleagues showed that chimeric transcripts found using NGS could also be detected at the protein level. Some chimeras incorporate signal peptides that could direct proteins to the ER and Golgi apparatus [58]. The Trim58/OR2T8 protein would be an interesting candidate for functional characterization.

### Chimeric Transcripts with loc727924

OR4N4 is highly and almost exclusively expressed in testis and displays chimeric transcripts with an upstream gene locus, loc727924. Originally defined by Rinn and Chang, loc727924 is a long non-coding RNA [64]. Because our analysis revealed that all chimeric transcripts start at a new, unannotated exon between exons 3 and 4 of loc717924, we propose that these chimeric transcripts have a different initiation site of transcription than the loc727924 transcript and that part of the loc727924 locus serves as a complex 5′UTR for OR4N4. Previous studies have shown that long non-coding RNAs can activate the expression of protein-coding genes in their immediate genomic neighborhood [65]. Various 5′ untranslated exons have been detected in human and mouse OR genes, especially in expressed transcripts of testis [9], [66], [67]. It is known that transcription of MOR23, the murine lyral receptor, is initiated from two distinct regions and that these are differentially utilized in the olfactory epithelium and testis [7]. The authors of that study postulated that different 5′UTR structures may be required for posttranscriptional regulation of the expression of this OR. Initiation of transcription of the OR4N4 gene in the human olfactory epithelium would be an interesting aspect to investigate to evaluate whether transcription of this gene is regulated in a novel way. It has been already postulated that the OR genes that are expressed in diverse tissues require different promoters for flexible transcriptional regulation, a feature that has already been demonstrated for other genes [9], [68].

### Internal Splicing

Previous analysis of mouse EST data suggested that splicing within the OR ORF can, for example, produce a protein with only five transmembrane domains [8]. Our investigation of internal splicing events revealed the existence of ORF splice variants of at least 3 out of the 20 most highly expressed ORs in some tissues; these splice variants do not encode complete OR proteins with seven transmembrane domains. For example, we showed that internal splicing within the ORF of OR4N4 occurs and that the number of transcripts of the complete ORF is therefore reduced. Due to a frame shift, splicing leads to premature termination after RNA coding for 90 amino acids has been transcribed. Due to premature stops or frame shifts, internal splicing within the broadly expressed OR51E1 transcript can also lead to a protein containing only the first OR transmembrane domain. Therefore, we think that in several of the cases analyzed in this study, internal splicing leads to the production of non-functional OR proteins. Relevant to this, it should be determined whether potential truncated OR proteins interfere with the function of complete OR proteins. The results of this study indicate that some ectopically OR transcripts may not code for a functional protein.

### Other Genes

In olfactory neurons, binding of an odorant to its OR activates a cAMP-mediated second messenger pathway consisting of Gα_olf_, adenylyl cyclase III and CNG-channels. As Plutznick and colleagues have already shown using mouse kidney, expression of these major elements is not restricted to primary chemosensory cells [27]. Although most of the basic components of the cAMP-mediated pathway were detected in all tissues, the specific subunit CNGA2 of CNG channels is expressed only in testis, an indication that a potential olfactory signal transduction pathway targets effectors other than cAMP-gated ion channels. Spehr and coauthors suggested that activation of OR51E2 by ß-ionone leads to Src kinase-dependent influx of Ca^2+^ ions via TRPV6 channels in prostate cells [69]. Furthermore, odorant-induced signaling was shown to activate phospholipase C in gastrointestinal enterochromaffin cells [18]. The results indicate that activation of ectopically expressed ORs may target signaling pathways other than those involved in classical olfactory signal transduction.

Our data also present a comprehensive overview of the widespread ectopic expression of non-olfactory chemoreceptors in various human tissues. The VNO-type chemoreceptor VN1R1, the function of which in humans is still elusive, is present in human olfactory epithelium, brain, kidney, liver and lung [70], [71]. We were able to show that VN1R1 is also expressed in nearly all of the human tissues investigated. Furthermore, we detected the sweet taste receptor Tas1R3 and the bitter taste receptors Tas2R14 and Tas2R20 in all 16 investigated tissues. Interestingly, Tas2R14 is a broad-range bitter receptor that responded to 33 of 104 tested bitter substances [72] and that could be responsible for the detection of bitter substances throughout the human body. Several studies have addressed the ectopic expression of taste receptors, for example in the gastrointestinal tract, in airways and in mammalian spermatozoa [73]–[76]. In human gut, Tas1R3 is involved in the glucose-stimulated secretion of glucagon-like peptide-1 [77]. Our study supports previous data indicating that non-olfactory chemoreceptors are expressed in a variety of human tissues. The results obtained using RNA-Seq provide a brief but comprehensive overview of the expression of taste receptors and vomeronasal receptors.

### Conclusion

In summary, recent advances in deep sequencing technologies have allowed us to conduct the first comprehensive RNA-Seq analysis of ectopically expressed ORs using a broad panel of human tissues. One hundred and eleven OR genes were found to be expressed in the investigated tissues. The expression pattern of several ORs is highly conserved, and many ORs are broadly expressed in various tissues. However, some ORs are tissue-specific. A possible explanation for the broad ectopic expression of some ORs is the fusion of transcripts with transcripts of upstream genes. Our results support the hypothesis that ORs play a functional role not only in the olfactory system but also in many other tissues.

## Methods

### Alignment of RNA-Seq Reads using TopHat

The Body Map 2.0 data used in this study were obtained with the Illumina Genome Analyzer HiSeq2000 (read length: 75 bp and 50 bp paired-end). For each tissue, standard mRNA-Seq libraries were prepared from poly-A selected mRNA. Samples were not multiplexed. Data were obtained from the NCBI GEO database with the accession number GSE30611. Data from Wang and colleagues were obtained using an Illumina Genome Analyzer (read length: 32 bp), and library preps were made according to standard protocols (for further information, see: [36]; GSE12946).

We analyzed the sequence data as previously described [78]. RNA-Seq reads were aligned to the hg19 reference genome by TopHat v1.2.0 [79]. The TopHat aligner is open-source software that can identify splice junctions (http://tophat.cbcb.umd.edu). The software SAM tools sort and index files in the BAM format [80]. Aligned data were visualized with the Integrative Genomic Viewer (http://www.broadinstitute.org/igv/). The command line used while using TopHat was selected as follows:

tophat --output-dir <name output> --GTF <hg19refseq.gtf> <indexes> tissue.fq

### Alignment Assembly and Gene Expression using Cufflinks

The software Cufflinks v1.0.3 was used to calculate abundance of transcripts [81] on the base of the refseq gene model. The reference transcriptome (hg19) is available in Gene Transfer Format (GTF) from the UCSC Genome Bioinformatics site of the University of California Santa Cruz and was modified to include also all OR pseudogenes that were lacking in the reference data but are listed in the HORDE database. The accuracy of the relative transcript abundance estimation was improved with a multifasta file (hg19.fa) [82]. The relative abundance of transcripts was reported in FPKM (fragments per kilobase of exon per million fragments mapped) units [81]. The Cufflinks parameters are listed below.

cufflinks --output-dir <name output2> --GTF <hg19refseq.gtf> --multi-read-correct --compatible-hits-norm --min-frags-per-transfrag 1 --frags-bias-correct <hg19.fa> sorted.bam

### RNA-Seq Background Estimation

To estimate an FPKM value to use in designating a gene as expressed, we used the approach described by Ramsköld et al. in 2009 [32]. A collection of intergenic regions were used to estimate the technical background over which genes can be classified with high confidence as expressed. Referring to van Bakel et al. [83], suitable intergenic regions do not lie within introns or 10 kb down- or upstream of a gene. Annotated genes from several gene models available from the UCSC table browser (Vega genes, UCSC, tRNA, snomiRNA, refseqgenes, NScanGenes, lincRNA, GenidGene, GenecodeV12, Ensembl, AceGene) were considered. We used a perl script to construct a gtf file in which the number and length distribution of intergenic regions were equal to the annotated exons of the refseq gene model but were spread randomly across intergenic parts of the genome. After setting intergenic regions, we calculated FPKM values with Cufflinks for these regions for every Body Map data set, thus obtaining a background distribution for each analyzed data set. A comparison between the expression levels of exons and intergenic regions of all tissues together was then used to find a threshold for detectable expression above background (Figure S3). In the manner described by Ramsköld, we set the threshold value at 0.1 FPKM, which is slightly higher than the intersection of the false discovery rate and the false negative rate (Figure S3). Approximately 44% of the detected ORs have FPKM values lower than this threshold. To estimate the true number of expressed genes, we multiplied the detected refseq genes by the false discovery rate for each bin. The subtraction of this product from the number of detected genes in each bin, revealed the true number of expressed genes. The resulting curve (black) was compared to the detected refseq genes (red), same shown as in Fig S3A (Fig. S3C). Because we were also interested in low abundance RNA (FPKM<0.1), we calculated the fraction of true positive hits in the FPKM range between 0.01 and 0.1 FPKM. Within this range, we estimated the number of hits in randomly intergenic regions (false positives) and divided it by the number of expressed refseq genes (false and true positives). We found out that 16.5% of the detected genes of the Body Map data sets are false positive and concluded that 83.5% of the expressed genes within this range are true positives. Therefore, these genes were included in the analysis as a separate fraction of potentially expressed OR genes.

All bioinformatic analyses were run on a Linux-based computer. Further processing of data was carried out using Excel 2007, Sigma Plot 8.0 and Corel Draw X4.

To determine whether single read and paired-end data sets of the same tissue detected the same ORs, we compared both runs of thyroid tissue of the Body Map project. We found identical most highly expressed ORs (Figure S6) and a strong correlation of expression (R^2^ = 0.97).

### Total RNA and cDNA Synthesis

RNA samples were purchased from commercial sources (colon, brain, breast, kidney, and lung from BioChain Institute, Inc., Newark, NJ, USA and testis from Cell Applications, San Diego, CA, USA). The RNA samples were subjected to DNaseI-treatment using the TURBO DNA-free Kit (Life Technologies, Carlsbad, CA, USA) according to the standard protocol. cDNA synthesis was performed using the iScript cDNA Synthesis Kit (Bio-Rad Laboratories, Hercules, CA, USA) according to the manufacturer’s instructions. An equivalent of 50 ng of total RNA was used for each RT-PCR experiment.

### RT-PCR

To validate the expression of different ORs, we designed primers that detect ∼100–300 bp of the OR ORF (Figure S11). To detect multiple splice forms, we designed exon-exon spanning primers (Figure S11). PCR was performed using GoTaq qPCR Master Mix (Promega, Madison, WI, USA) with the Mastercycler realplex^2^ (eppendorf, Hamburg, Germany) (20 µl total volume, 40 cycles: 95°C, 59°C, 72°C, 45 s each). All experiments were conducted in triplicate.

## Supporting Information

Figure S1 **Expression patterns of housekeeping genes in different tissues.** The highly expressed (ß-actin (ACTB) and glyceraldehyde 3-phosphate dehydrogenase (GAPDH)), moderately expressed (ribosomal protein L29 (RPL29) and ribosomal protein L13A (RPL13A)) and weakly expressed genes (β-glucuronidase (GUSB), transferrin receptor (TFRC), hypoxanthine phosphoribosyltransferase 1 (HPRT1) and TATA box binding protein (TBP)) are frequently used as quantitative RT-PCR standards.
(PDF)

Figure S2 **Distribution of FPKM values in brain.** To obtain an estimate of FPKM values for the expression of genes, we calculated a histogram of FPKM distribution for brain tissue (Body Map 2.0). Values <0.3 can be regarded as indicating very weakly expressed, 0.3–3 as indicated weakly expressed and 3 and −30 as indicating moderately expressed genes. Values of 30–100 indicates high expression, and values >100 indicate extremely high expression. Of the ∼23000 analyzed genes, expression at >0.1 FPKM was detected for ∼17000 genes; mRNA for ∼500 of these genes is highly abundant, with FPKM >100.
(PDF)

Figure S3 **Estimation of an expression threshold.** **A:** Reads were mapped to refseq genes (red) and intergenic background regions (blue). The intergenic regions have the same length distribution as the exons of annotated refseq genes. The expression levels of all genes and background regions of all 16 Body Map tissues were binned. The figures focus on the expression effect between 0.01 and 10 FPKM. **B:** Bins were converted to cumulative amounts of expressed genes above each expression level (cumulative genes; dark red) and intergenic regions (cumulative intergenic; dark blue). A false discovery rate (FDR; green) was calculated at each expression level as described by Ramsköld et al. (2009). **C:** The true number of expressed genes in each bin (Approx true; black) was estimated from the observed numbers of refseq genes (red, same as A) by multiplication by the FDR. The genes expressed at levels between 0.01 and 0.1 FPKM are false positive in 16.5% of cases, whereas 83.5% of the genes within this range are true positive. The true number of expressed genes in each bin was converted to the cumulative amount, and the false negative rate (FNR) was estimated as described by Ramsköld et al. (2009). **D:** FDR and FNR for the detection of expressed genes as a function of the detection threshold used.
(PDF)

Figure S4 **Reliability of weakly expressed ORs (0.01–0.1 FPKM) in RNA-Seq data sets of testis. A:** We detected 51 ORs in the testis 75-bp single-read data set that showed expression in the range 0.01–0.1 FPKM. Of these ORs, 67% were also detected in the independent paired-end testis data set, indicating that most of these OR transcripts are true positive. **B:** In contrast, we detected only 6% of these ORs in the skeletal muscle data set, demonstrating that weakly expressed ORs are not derived from randomly distributed mapped reads.
(PDF)

Figure S5 **The sum of FPKM values of ORs per tissue.** The cumulative expression (the sum of FPKM values >0.01) of ORs and OR pseudogenes in various tissues is shown. The expression of ORs is more pronounced in testis than in any other tissue.
(PDF)

Figure S6 **Correlation of FPKM values of ORs between thyroid-sequencing 1×75 bp single-read data versus 2×50 bp paired-end data.** ORs with FPKM values >0.01 are shown. R^2^ is the coefficient of determination.
(PDF)

Figure S7 **Validation of the Trim58/OR2W3 chimeric transcript by RT-PCR.** The detected chimeric transcripts were confirmed by RT-PCR with a forward primer located in exon 3, 4 or 5 of the Trim58 gene and a reverse primer located in the ORF of OR2W3. We confirmed the amplified PCR products by Sanger sequencing. The double band in lane 1 represents splice variants. The upper band consists of exons 3, 4, 5 and parts of exon 6 of Trim58 and OR2W3. The lower band consists of the same components, except for exon 6 of Trim58. In lane 3, the weak upper band contains exon 6 of Trim58, while the lower band does not.
(PDF)

Figure S8 **OR13E1P is located within a cluster of highly expressed genes in brain tissue.** Sample representation of read coverage of an OR located in a highly expressed gene cluster (Integrative Genomic Viewer). The gray segments indicate reads that were mapped onto the reference genome. The transcript is indicated by blue bars (exon) or lines (intron). Above, the read coverage is shown (detected and mapped counts/bases at each respective position).
(PDF)

Figure S9 **Dependence of OR expression on the genomic neighborhood.** The bar diagram shows the dependence of the ectopic expression of ORs and OR on a non-OR neighbor.
(PDF)

Figure S10 **Validation of NGS-data by RT-PCR.** Comparison of RNA-Seq data with RT-PCR experiments. M = 50 bp DNA ladder; + = cDNA; − = RNA. PCR results were verified by Sanger sequencing. In some cases, the primers amplified fragments that originated from two ORs. In these cases, both names are given in column one.
(PDF)

Figure S11 **Primer sequences used for PCR and chimeric transcript validation.** The listed primers are shown in the 5′-3′ direction.
(PDF)

Figure S12 **Expression of the most highly ectopically expressed ORs in the human olfactory epithelium.** Out of the 40 most highly ectopically expressed ORs (RNA-Seq), 73% were detected in the human olfactory epithelium (microarray data [29]). The other most highly ectopically expressed ORs were not included or were not detectable in the previous microarray analysis of the olfactory epithelium.
(PDF)

Table S1 Expression profile of all genes in 16 different human tissues.
(XLSX)
