# Supplementary figures and images for: Expression Profile of Ectopic Olfactory Receptors Determined by Deep Sequencing
Source: PLoS One. 2013 Feb 6;8(2):e55368. doi: 10.1371/journal.pone.0055368 (PMC3566163; doi:10.1371/journal.pone.0055368)

Analysis of housekeeping gene expression by FPKM values

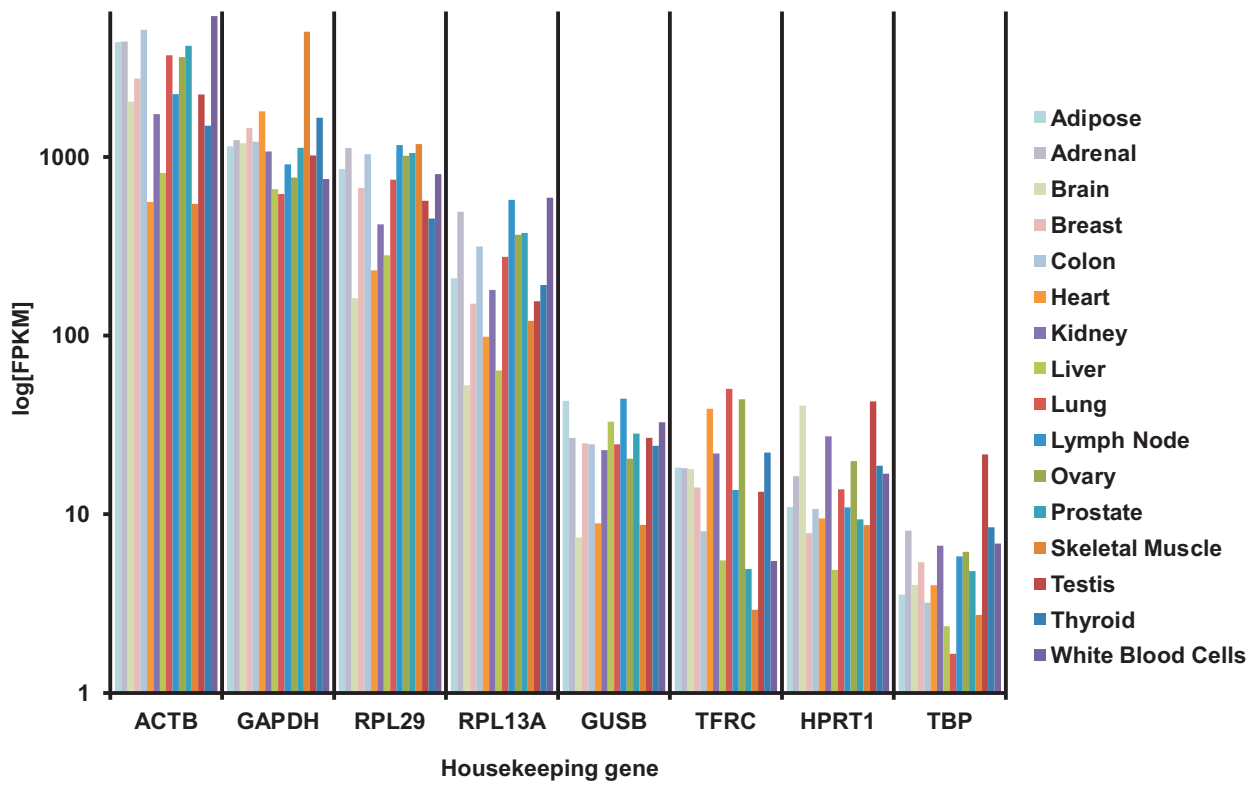

Supplement: Figure S1 — Expression patterns of housekeeping genes in different tissues. The highly expressed (ß-actin (ACTB) and glyceraldehyde 3-phosphate dehydrogenase (GAPDH)), moderately expressed (ribosomal protein L29 (RPL29) and ribosomal protein L13A (RPL13A)) and weakly expressed genes (β-glucuronidase (GUSB), transferrin receptor (TFRC), hypoxanthine phosphoribosyltransferase 1 (HPRT1) and TATA box binding protein (TBP)) are frequently used as quantitative RT-PCR standards. (PDF) [file pone.0055368.s001.pdf]

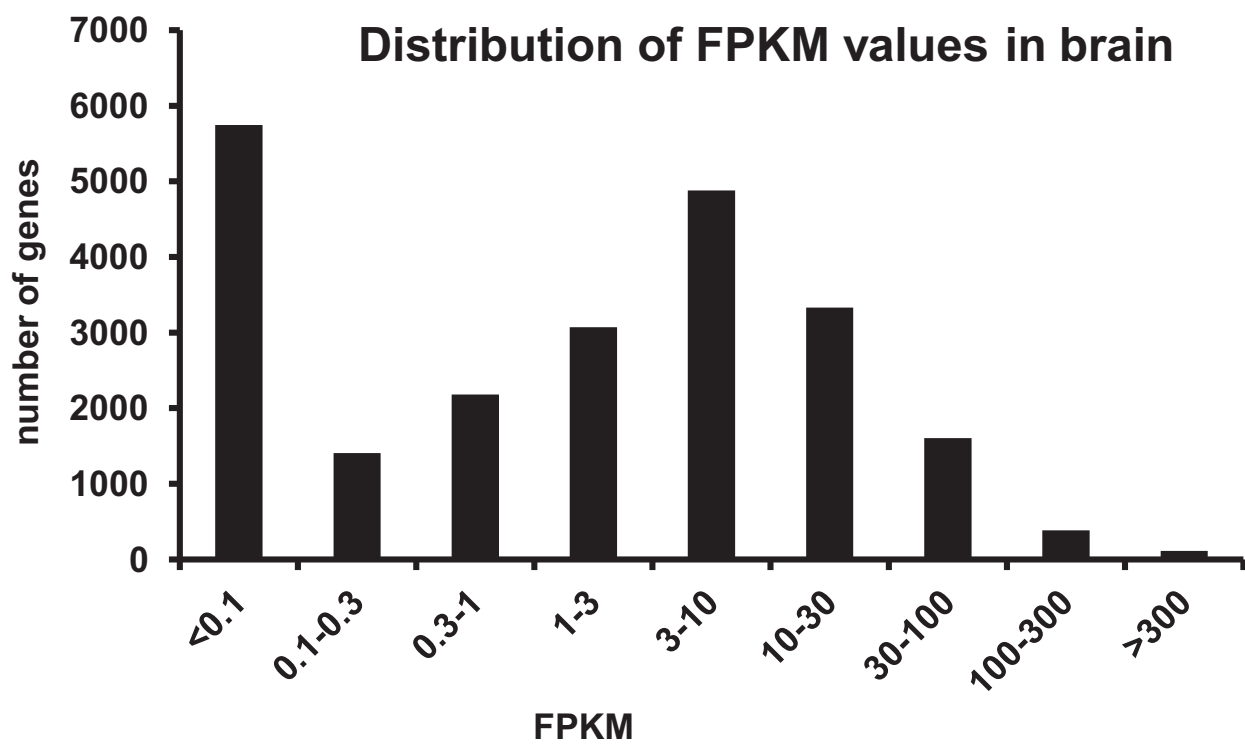

Supplement: Figure S2 — Distribution of FPKM values in brain. To obtain an estimate of FPKM values for the expression of genes, we calculated a histogram of FPKM distribution for brain tissue (Body Map 2.0). Values <0.3 can be regarded as indicating very weakly expressed, 0.3–3 as indicated weakly expressed and 3 and −30 as indicating moderately expressed genes. Values of 30–100 indicates high expression, and values >100 indicate extremely high expression. Of the ∼23000 analyzed genes, expression at >0.1 FPKM was detected for ∼17000 genes; mRNA for ∼500 of these genes is highly abundant, with FPKM >100. (PDF) [file pone.0055368.s002.pdf]

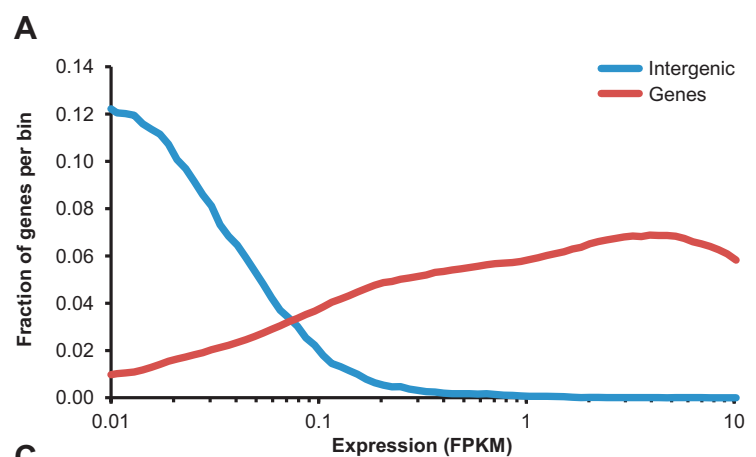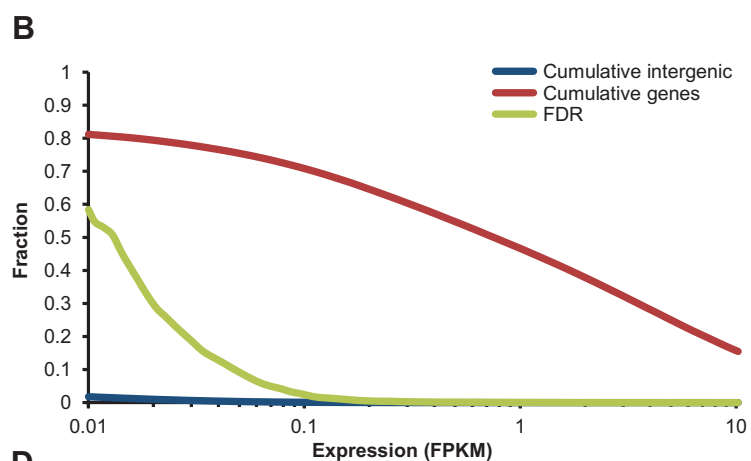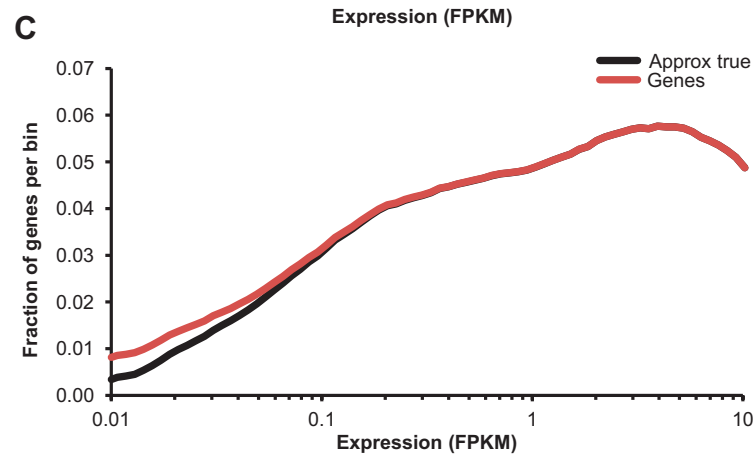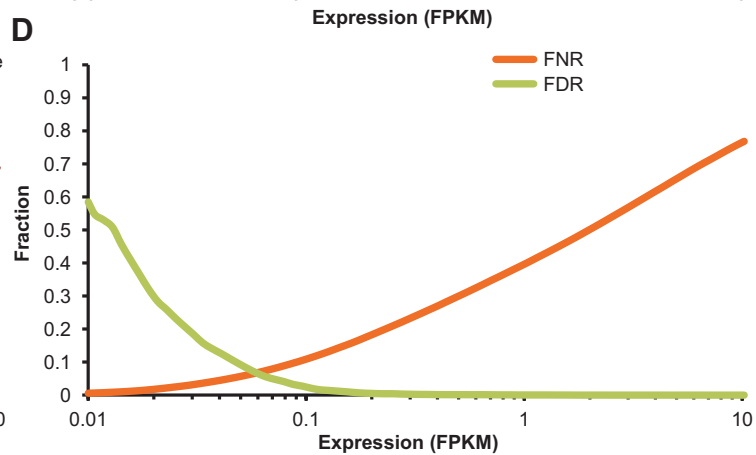

Supplement: Figure S3 — Estimation of an expression threshold. A: Reads were mapped to refseq genes (red) and intergenic background regions (blue). The intergenic regions have the same length distribution as the exons of annotated refseq genes. The expression levels of all genes and background regions of all 16 Body Map tissues were binned. The figures focus on the expression effect between 0.01 and 10 FPKM. B: Bins were converted to cumulative amounts of expressed genes above each expression level (cumulative genes; dark red) and intergenic regions (cumulative intergenic; dark blue). A false discovery rate (FDR; green) was calculated at each expression level as described by Ramsköld et al. (2009). C: The true number of expressed genes in each bin (Approx true; black) was estimated from the observed numbers of refseq genes (red, same as A) by multiplication by the FDR. The genes expressed at levels between 0.01 and 0.1 FPKM are false positive in 16.5% of cases, whereas 83.5% of the genes within this range are true positive. The true number of expressed genes in each bin was converted to the cumulative amount, and the false negative rate (FNR) was estimated as described by Ramsköld et al. (2009). D: FDR and FNR for the detection of expressed genes as a function of the detection threshold used. (PDF) [file pone.0055368.s003.pdf]

**A**

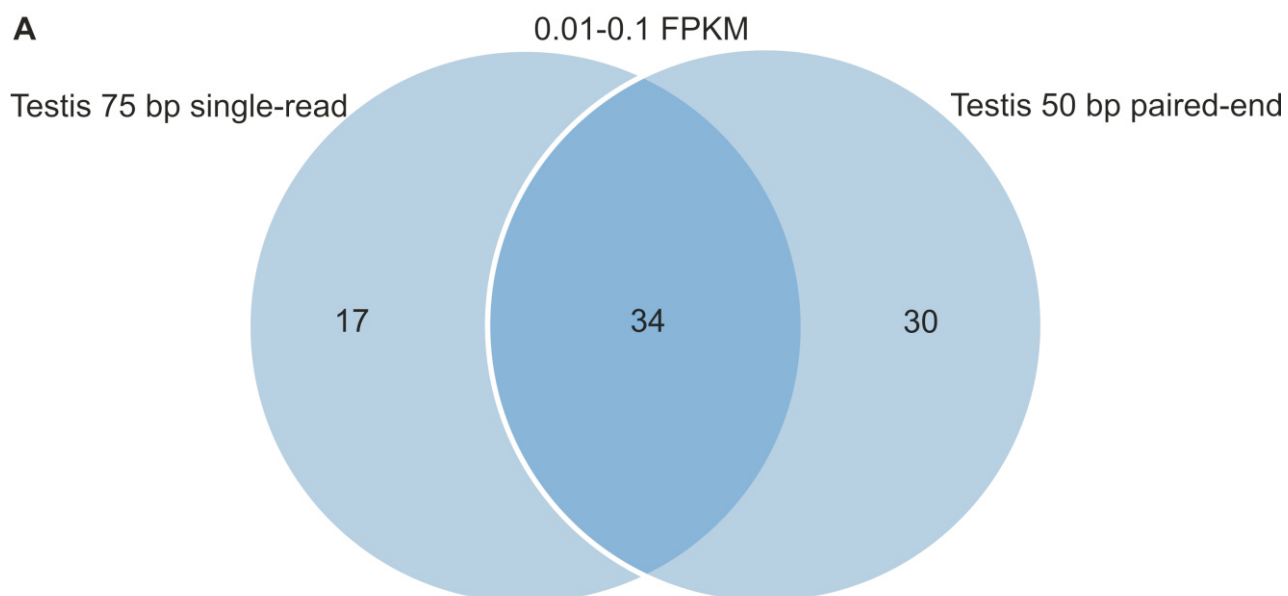

**B**

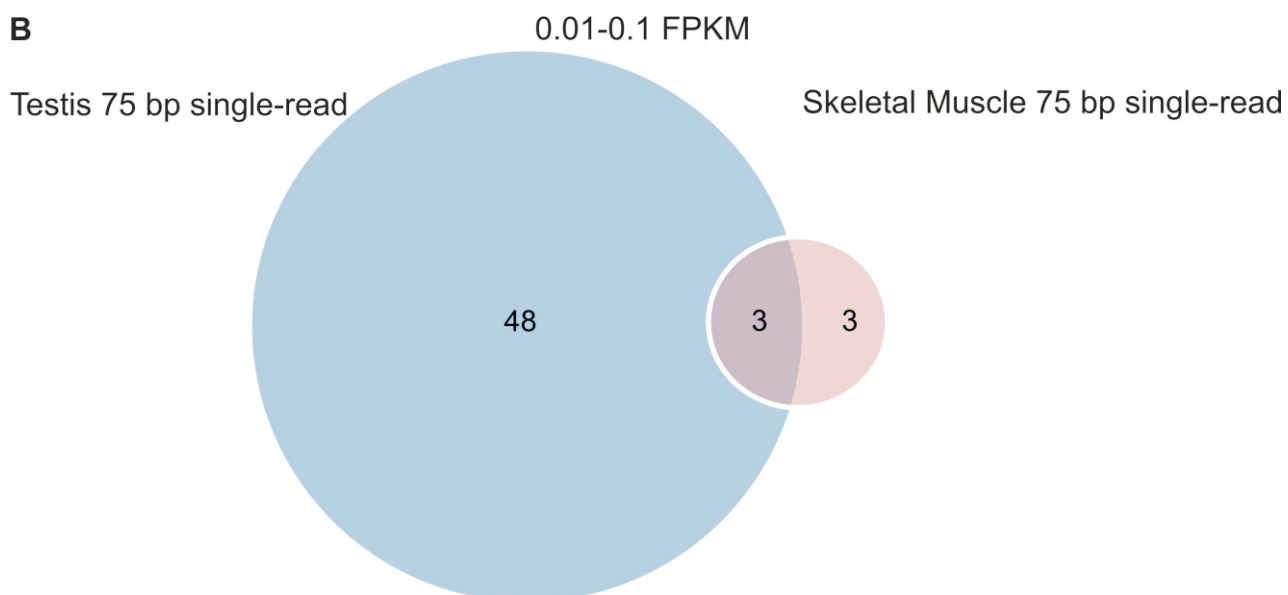

Supplement: Figure S4 — Reliability of weakly expressed ORs (0.01–0.1 FPKM) in RNA-Seq data sets of testis. A: We detected 51 ORs in the testis 75-bp single-read data set that showed expression in the range 0.01–0.1 FPKM. Of these ORs, 67% were also detected in the independent paired-end testis data set, indicating that most of these OR transcripts are true positive. B: In contrast, we detected only 6% of these ORs in the skeletal muscle data set, demonstrating that weakly expressed ORs are not derived from randomly distributed mapped reads. (PDF) [file pone.0055368.s004.pdf]

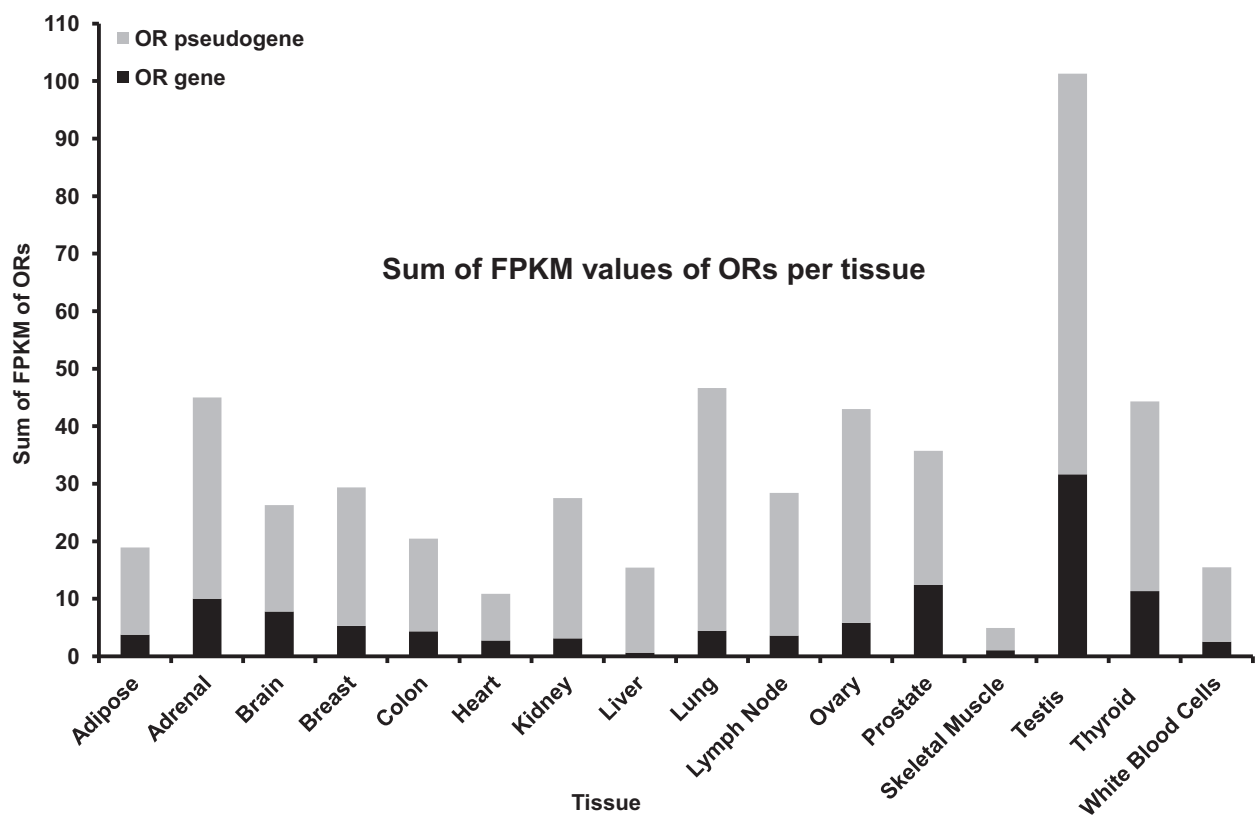

Supplement: Figure S5 — The sum of FPKM values of ORs per tissue. The cumulative expression (the sum of FPKM values >0.01) of ORs and OR pseudogenes in various tissues is shown. The expression of ORs is more pronounced in testis than in any other tissue. (PDF) [file pone.0055368.s005.pdf]

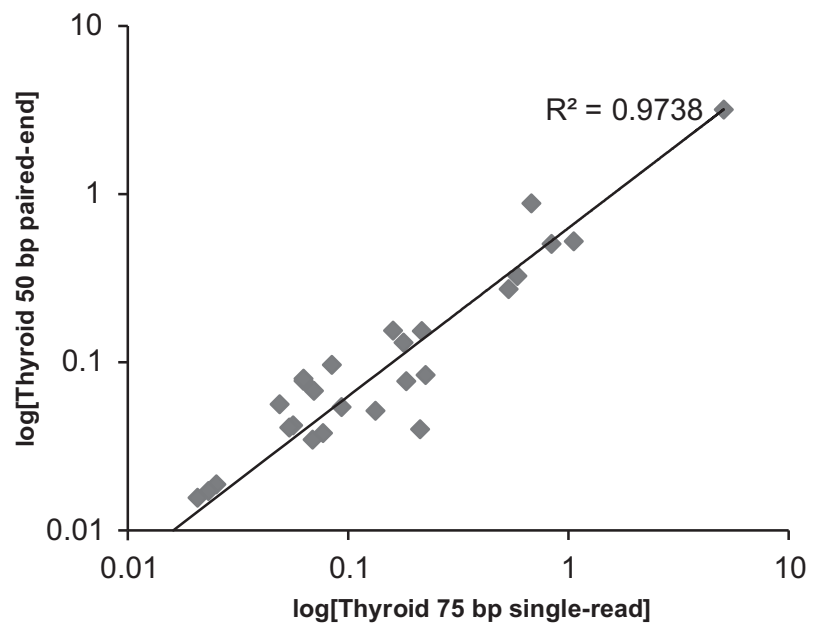

Supplement: Figure S6 — Correlation of FPKM values of ORs between thyroid-sequencing 1×75 bp single-read data versus 2×50 bp paired-end data. ORs with FPKM values >0.01 are shown. R2 is the coefficient of determination. (PDF) [file pone.0055368.s006.pdf]

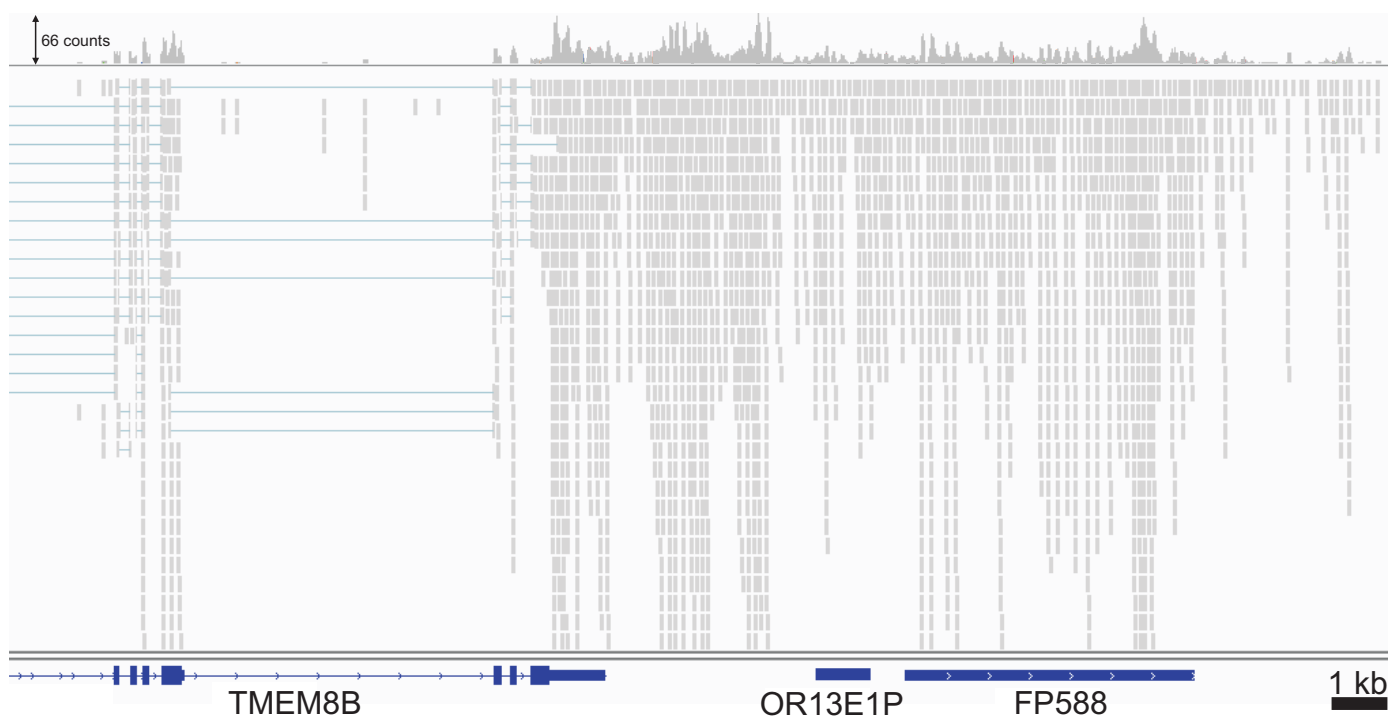

Supplement: Figure S8 — OR13E1P is located within a cluster of highly expressed genes in brain tissue. Sample representation of read coverage of an OR located in a highly expressed gene cluster (Integrative Genomic Viewer). The gray segments indicate reads that were mapped onto the reference genome. The transcript is indicated by blue bars (exon) or lines (intron). Above, the read coverage is shown (detected and mapped counts/bases at each respective position). (PDF) [file pone.0055368.s008.pdf]

**Dependence of OR expression on the genomic neighborhood**

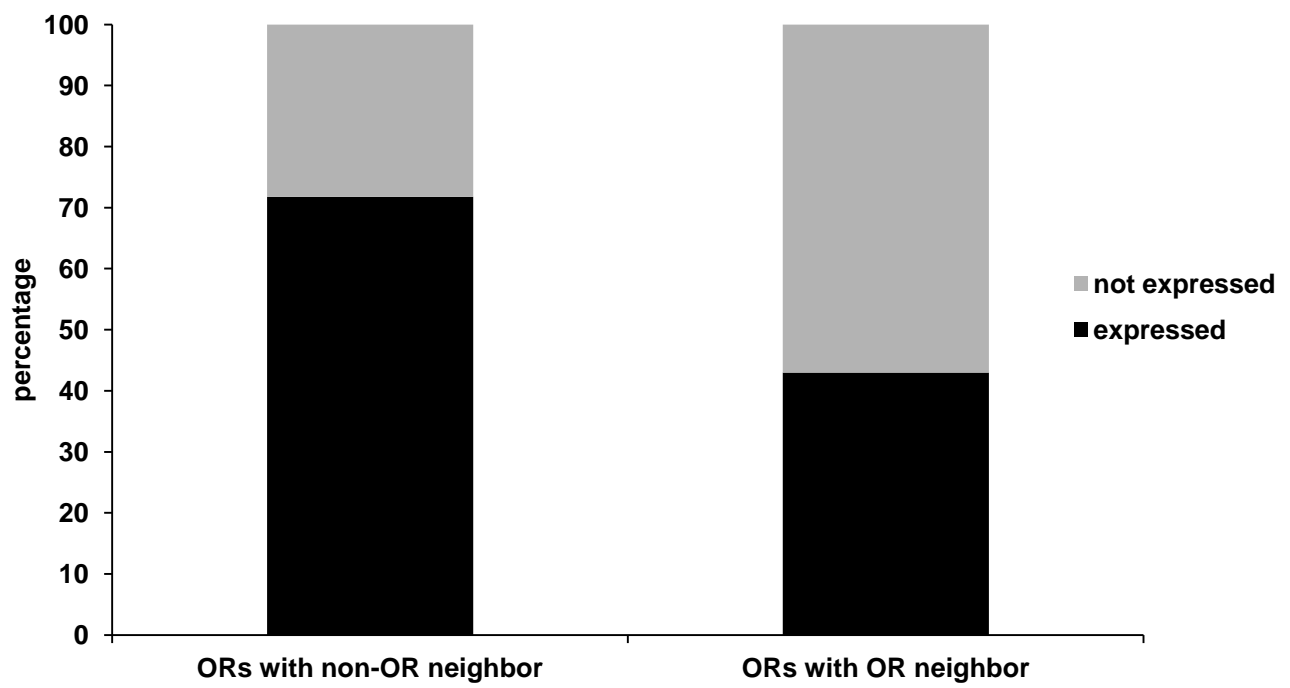

Supplement: Figure S9 — Dependence of OR expression on the genomic neighborhood. The bar diagram shows the dependence of the ectopic expression of ORs and OR on a non-OR neighbor. (PDF) [file pone.0055368.s009.pdf]

## The 40 most highly ectopically expressed ORs

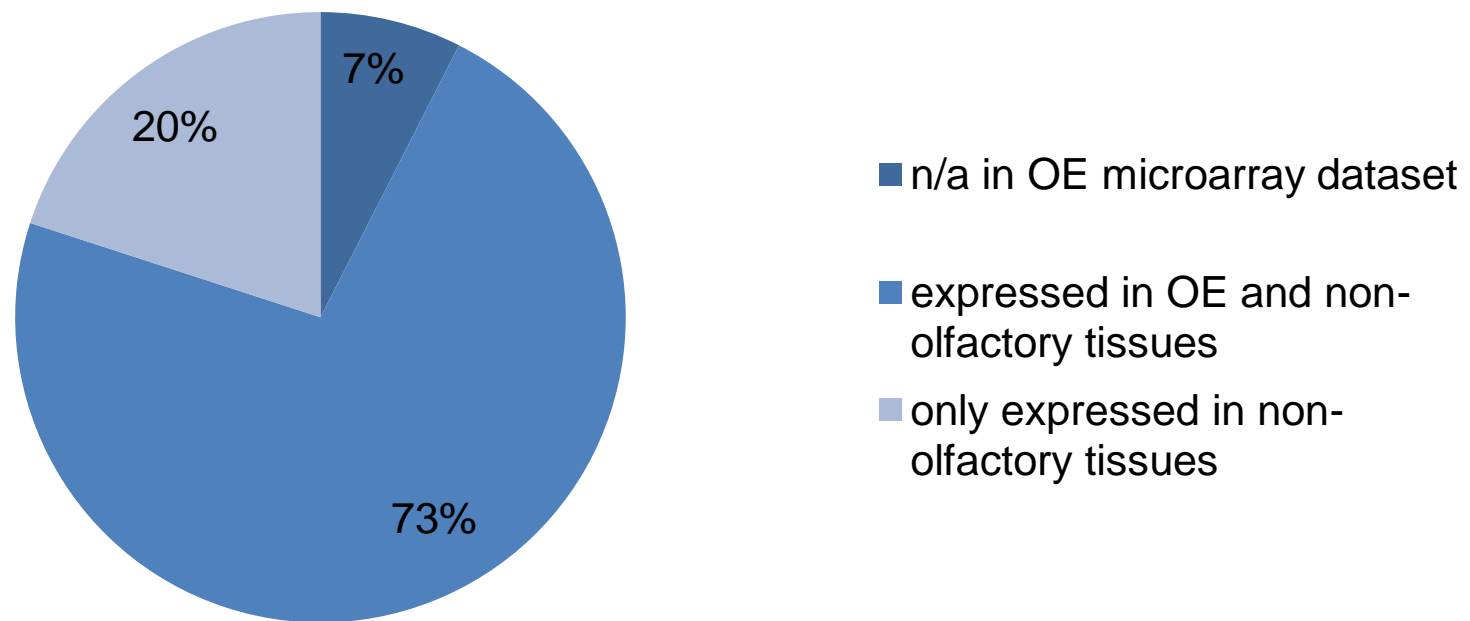

Supplement: Figure S12 — Expression of the most highly ectopically expressed ORs in the human olfactory epithelium. Out of the 40 most highly ectopically expressed ORs (RNA-Seq), 73% were detected in the human olfactory epithelium (microarray data [29]). The other most highly ectopically expressed ORs were not included or were not detectable in the previous microarray analysis of the olfactory epithelium. (PDF) [file pone.0055368.s012.pdf]
