# Supplementary material for: Expression Profile of Ectopic Olfactory Receptors Determined by Deep Sequencing
Source: PLoS One. 2013 Feb 6;8(2):e55368. doi: 10.1371/journal.pone.0055368 (PMC3566163; doi:10.1371/journal.pone.0055368)
Supplement: Figure S7 — Validation of the Trim58/OR2W3 chimeric transcript by RT-PCR. The detected chimeric transcripts were confirmed by RT-PCR with a forward primer located in exon 3, 4 or 5 of the Trim58 gene and a reverse primer located in the ORF of OR2W3. We confirmed the amplified PCR products by Sanger sequencing. The double band in lane 1 represents splice variants. The upper band consists of exons 3, 4, 5 and parts of exon 6 of Trim58 and OR2W3. The lower band consists of the same components, except for exon 6 of Trim58. In lane 3, the weak upper band contains exon 6 of Trim58, while the lower band does not. (PDF) [file pone.0055368.s007.pdf]

OR2W3 - Trim58 in lung

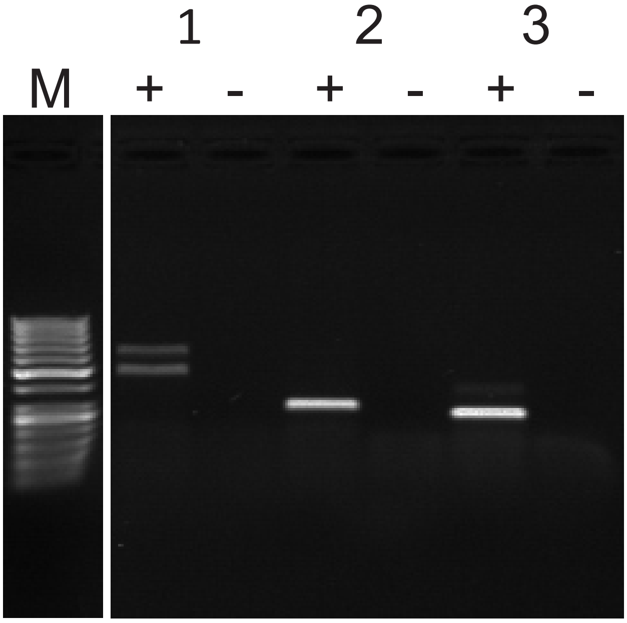

1: fwd primer in exon 3 of Trim58
rv primer in OR2W3
2: fwd primer in exon 4 of Trim58
rv primer in OR2W3
3: fwd primer in exon 5 of Trim58
rv primer in OR2W3
