# Supplementary material for: Expression Profile of Ectopic Olfactory Receptors Determined by Deep Sequencing
Source: PLoS One. 2013 Feb 6;8(2):e55368. doi: 10.1371/journal.pone.0055368 (PMC3566163; doi:10.1371/journal.pone.0055368)
Supplement: Figure S10 — Validation of NGS-data by RT-PCR. Comparison of RNA-Seq data with RT-PCR experiments. M = 50 bp DNA ladder; + = cDNA; − = RNA. PCR results were verified by Sanger sequencing. In some cases, the primers amplified fragments that originated from two ORs. In these cases, both names are given in column one. (PDF) [file pone.0055368.s010.pdf]

OR		M 50 bp	Brain	Breast	Colon	Kidney	Lung	Testis
			+ -	+ -	+ -	+ -	+ -	+ -
1C1	250 bp	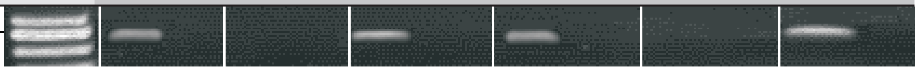						
	FPKM		0	0	0	0	0	1.09
1E1	250 bp	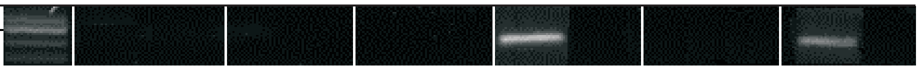						
	FPKM		0	0.31	0	0	0	0.1
1F1	300 bp	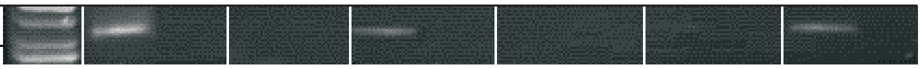						
	FPKM		0.39	0	0	0	0	0.1
1L8	250 bp	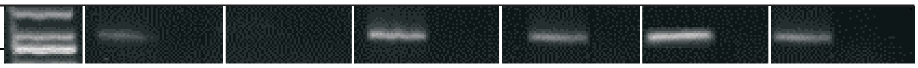						
	FPKM		0.27	0	0	0.10	0	0.15
2A1/ 2A42	250 bp	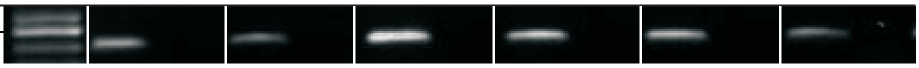						
	FPKM		0	0.29	0.35	0.31	0.43	0.18
2A4/ 2A7	250 bp	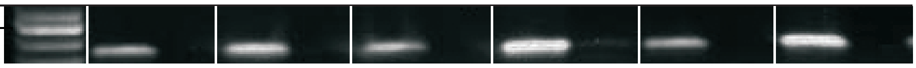						
	FPKM		0	0.12	0.12	0.52	0.16	0.16
2C3	250 bp	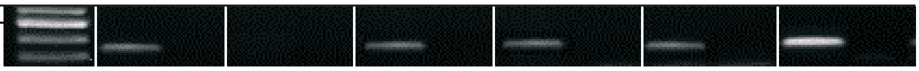						
	FPKM		0	0.14	0	0	0	0.49
2H1/ 2H2	250 bp	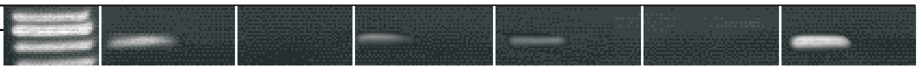						
	FPKM		0/ 0.25	0/ 0	0/ 0	0/ 0	0/ 0	1.04/ 0.51
2K2	250 bp	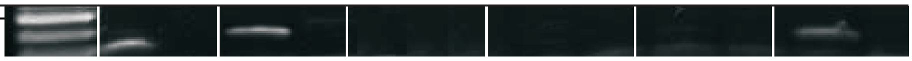						
	FPKM		0.14	0.10	0	0	0	0.29
2L13	200 bp	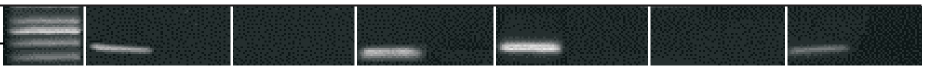						
	FPKM		0.65	0	0.24	0	0	0
2W3	200 bp	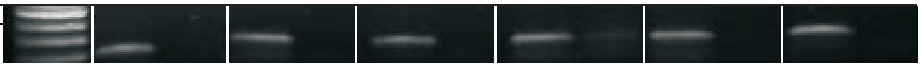						
	FPKM		0.42	0	0.10	0	1.95	1.04
3A2/ 3A3	250 bp	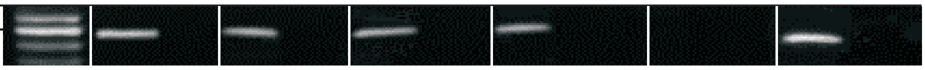						
	FPKM		0.11/ 0.12	0.18/ 0.19	0/ 0	0/ 0	0/ 0	1.12/ 0.36

OR		M 50 bp	Brain	Breast	Colon	Kidney	Lung	Testis
			+ -	+ -	+ -	+ -	+ -	+ -
4N4/ 4N3P	250 bp	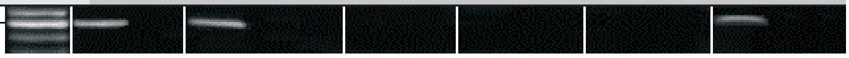						
	FPKM		0.02/ 0.02	0/ 0	0/ 0	0/ 0	0/ 0	9.85/ 1.62
5K2	250 bp	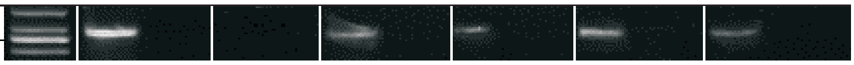						
	FPKM		0.1	0.21	0.1	0.1	0.04	0
7A5	250 bp	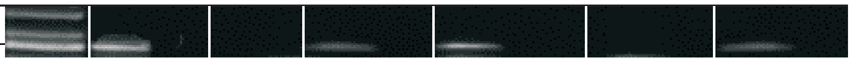						
	FPKM		0.30	0.1	0.02	0	0	0.28
7C1	300 bp	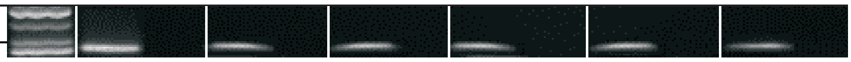						
	FPKM		0.13	0	0.04	0	0	0.70
8D1	250 bp	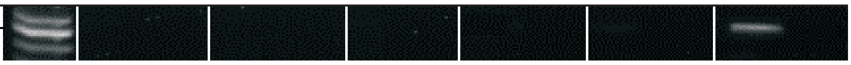						
	FPKM		0	0.18	0	0	0	0.58
10A2/ 10A5	250 bp	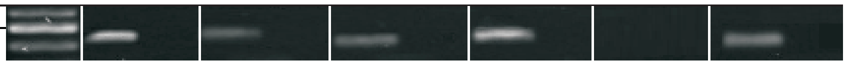						
	FPKM		0.16/ 0	0.13/ 0	0/ 0	0.13/ 0	0/ 0	0.1/ 0.06
10AD1	200 bp	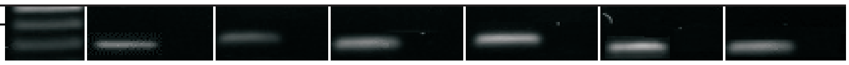						
	FPKM		0.25	0.1	0	0	0	0.17
13J1	250 bp	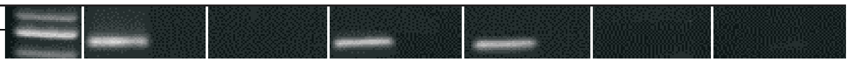						
	FPKM		0.64	0	0	0	0	0
51E1	250 bp	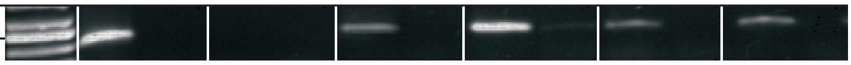						
	FPKM		0.15	1.72	0.12	0.55	0	0.28
51E2	250 bp	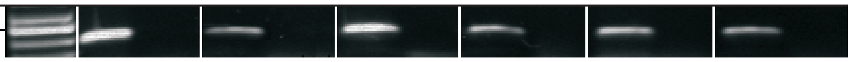						
	FPKM		0.04	0.28	2.36	0.26	0.15	0.17
52D1	150 bp	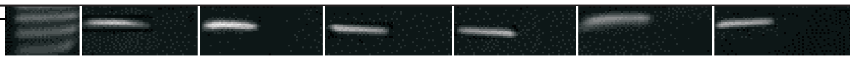						
	FPKM		0	0	0	0	0.04	0.53
52N4	250 bp	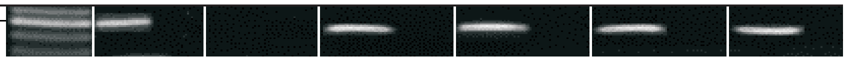						
	FPKM		0.03	0.03	0	0	0.34	0.17
56B1	250 bp	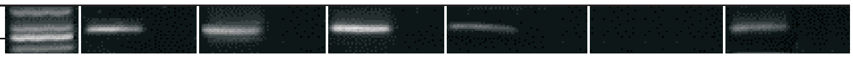						
	FPKM		0.08	0.06	0	0.13	0.06	0
