# Supplementary material for: Expression Profile of Ectopic Olfactory Receptors Determined by Deep Sequencing
Source: PLoS One. 2013 Feb 6;8(2):e55368. doi: 10.1371/journal.pone.0055368 (PMC3566163; doi:10.1371/journal.pone.0055368)
Supplement: Figure S11 — Primer sequences used for PCR and chimeric transcript validation. The listed primers are shown in the 5′-3′ direction. (PDF) [file pone.0055368.s011.pdf]

Gene	Primer Sequence	Product Size (bp)
OR10A2/5 fwd	CCCTGCTTGCCCAGGACACA	200
OR10A2/5 rev	GAACCAGGAGGCAGCAGCCA	
OR10AD1 fwd	ACTGCCTGGTTCTTTGGGCTGA	120
OR10AD1 rev	GCCAATCACTATGGGGGCCTCA	
OR13J1 fwd	CTGGCCATCTGCCAGCCACT	206
OR13J1 rev	GTGTTGCCGCATGCCAGCTT	
OR1C1 fwd	TTTGCAGGCTGCCTCACCCA	197
OR1C1 rev	TGCAGGAGGGCGTGGAGGTA	
OR1E1 fwd	CCATCCCCTATGCGGACTGCC	188
OR1E1 rev	GGTGGTCAGCACCCAGGACAG	
OR1F1 fwd	GGCCACTGTCCTGGGGAACC	275
OR1F1 rev	GGCACACGGCGACAAAGTGG	
OR1L8 fwd	GGCCATTCGCTTCAACCCCC	298
OR1L8 rev	GGCCACCAGCAGGACACAGT	
OR2A1/42 fwd	CCTGCTCCTCCCACCTCTGC	199
OR2A1/42 rev	CCCAGTGCTCTCCTCAGGGC	
OR2A4/7 fwd	GGTGCCCCGGATGCTGGTG	157
OR2A4/7 rev	GGGGTGGCAGATGGCCACG	
OR2C3 fwd	CCCTTCCTGGACATGAGCTTCACC	151
OR2C3 rev	TGGCCAGCAGGACACACTCG	
OR2H1/2 fwd	GCCCAGGCAGTGCTGAGGAT	155
OR2H1/2 rev	AACTTGCCCCTCCCTTGGGC	
OR2K2 fwd	GTGTGTGCTCCTGGCCGTGAT	146
OR2K2 rev	TTTCCAGCAGAGCGGTCAGGC	
OR2L13 fwd	CGGTGGGTAACTCGGCCATGA	133
OR2L13 rev	TACGCCATCTTGGGGACGGTG	
OR2W3 fwd	CTGCCGGGGCTTGGTGTCAG	105
OR2W3 rev	TCCACCTCGTGGTGCCCACA	
OR3A2/3 fwd	TGGCAGCCGTCTTGGTGGAG	229
OR3A2/3 rev	CGGTCATAGGCCATGGCGGT	
OR4N3P/4 fwd	CCGCTTGCCTTTTTGTGGCCC	233
OR4N3P/4 rev	TGGTGCACGTGGACATGGCC	
OR51E1 fwd	CCGCCATTGGCCTGGACTCA	213
OR51E1 rev	CCAAGATGACGGGCAGCGGA	
OR51E2 fwd	CCCAGATTGGCATCGTGGCTG	201
OR51E2 rev	CGCCCATGACCAGCAGAATGG	
OR52D1 fwd	TCTGAGTACCTGTGGCTCCCAC	78
OR52D1 rev	CGGTGGGTGAGGAAGGAGAAGA	
OR52N4 fwd	CCAAGCTCCTGCCCTACTGC	226
OR52N4 rev	AAGGCCTTCTGCCGAGCATC	
OR56B1 fwd	GCCAAGGCCCTGAGCACTTGTA	222
OR56B1 rev	GGCAAACAGCACCTTTTGGAAGG	
OR5K2 fwd	AGATGACCACAGGCGCCTTCA	284
OR5K2 rev	GGCTTTGGCCCTTCCCTCCT	
OR7A5 fwd	CAGTTGCGCTGCTGGGTGGA	200
OR7A5 rev	TGAGTTGCGGGTGGCAGCAG	
OR7C1 fwd	TGAACCCCCAGCTCTGTGGAC	220
OR7C1 rev	ATCACACCCAGGACGCCAGTT	
OR7D2 fwd	ACCCCCACCTCTGTGGCCTC	216
OR7D2 rev	AAACGCCCAGCACACCCGTC	
OR8D1 fwd	ACTGCTAGTGCTGGCTGCCT	285
OR8D1 rev	GCTTTGGACCGGCCCTCTGA	

OR2A7/loc728377 fwd	TCCCTTCTCCTCCATTCGGGGG	409
OR2A7/loc728377 rev	TCGACGACCGCCAGGTGTGA	
OR2W3/Trim58 Ex3 fwd	AGTTTGAGAAGCATCGTGGC	
OR2W3/Trim58 Ex4 fwd	TGTGAGAGGAGTCCTGAGCAG	
OR2W3/Trim58 Ex5 fwd	GGCTGGAAGCAGAGAACATC	
OR2W3/Trim58 rev	AGGTACGCGATCAGGATGAC	
OR4N4/loc727924 fwd	GTCACAGACAGAACATTGGCACCT	500
OR4N4/loc727924 rev	TCCACCAACATCCTGGGAGCCA	
OR7E14P/Plekha7 fwd	CGGGGAGCCCGTCAACTCG	200
OR7E14P/Plekha7 rev	TGGACAGGAACAGCCCAGCGA	
